# Structural mutations of small single copy (SSC) region in the plastid genomes of five *Cistanche* species and inter-species identification

**DOI:** 10.1186/s12870-022-03682-x

**Published:** 2022-08-25

**Authors:** Yujing Miao, Haimei Chen, Wanqi Xu, Qiaoqiao Yang, Chang Liu, Linfang Huang

**Affiliations:** Key Laboratory of Chinese Medicine Resources Conservation, State Administration of Traditional Chinese Medicine of China, Institute of Medicinal Plant Development, Chinese Academy of Medical Sciences, Peking Union Medical College, Beijing, 100193 China

**Keywords:** *Cistanche*, Plastome, Structural variation, Gene loss, Pseudogenization, Molecular marker

## Abstract

**Background:**

*Cistanche* is an important genus of *Orobanchaceae*, with critical medicinal, economic, and desertification control values. However, the phylogenetic relationships of *Cistanche* genus remained obscure. To date, no effective molecular markers have been reported to discriminate effectively the *Cistanche* closely related species reported here. In this study, we obtained and characterized the plastomes of four *Cistanche* species from China, to clarify the phylogenetic relationship within the genus, and to develop molecular markers for species discrimination.

**Results:**

Four *Cistanche* species (*Cistanche deserticola*, *Cistanche salsa*, *Cistanche tubulosa* and *Cistanche sinensis*), were deep-sequenced with Illumina. Their plastomes were assembled using SPAdes and annotated using CPGAVAS2. The plastic genomes were analyzed in detail, finding that all showed the conserved quadripartite structure (LSC-IR-SSC-IR) and with full sizes ranging from 75 to 111 Kbp. We observed a significant contraction of small single copy region (SSC, ranging from 0.4–29 Kbp) and expansion of inverted repeat region (IR, ranging from 6–30 Kbp), with *C. deserticola* and *C. salsa* showing the smallest SSCs with only one gene (*rpl3*2). Compared with other *Orobanchaceae* species, *Cistanche* species showed extremely high rates of gene loss and pseudogenization, as reported for other parasitic *Orobanchaceae* species. Furthermore, analysis of sequence divergence on protein-coding genes showed the three genes (*rpl*22, *clp*P and *ycf*2) had undergone positive selection in the *Cistanche* species under study. In addition, by comparison of all available *Cistanche* plastomes we found 25 highly divergent intergenic spacer (IGS) regions that were used to predict two DNA barcode markers (Cis-mk01 and Cis-mk02 based on IGS region *trn*R-ACG-*trn*N-GUU) and eleven specific DNA barcode markers using Ecoprimer software. Experimental validation showed 100% species discrimination success rate with both type of markers.

**Conclusion:**

Our findings have shown that *Cistanche* species are an ideal model to investigate the structure variation, gene loss and pseudogenization during the process of plastome evolution in parasitic species, providing new insights into the evolutionary relationships among the *Cistanche* species. In addition, the developed DNA barcodes markers allow the proper species identification, ensuring the effective and safe use of *Cistanche* species as medicinal products.

**Supplementary Information:**

The online version contains supplementary material available at 10.1186/s12870-022-03682-x.

## Background

*Cistanche* species are a group of non-photosynthetic parasitic plants, belonging to the *Orobanchaceae*. The family contains nonparasitic, hemiparasitic as well as holoparasitic species [1]. The *Orobanchaceae* are an excellent model system for: a) the studying of plant parasitism evolution; b) the investigation of phenotypic plasticity [2, 3]. *Cistanche* species, as nonphotosynthetic holoparasites, are completely devoid of chlorophyll and functional leaves. They are remarkable in appearance, comprising imposing, brightly colored flowering spikes that sprout seemingly from bare earth, which has earned them their common name ‘desert hyacinths’ [4]. Some 20–30 accepted species known as the Old World constitute the genus *Cistanche*, which traverses from the west (Macaronesia) to the east (Northwest China) [4]. *Cistanche* species grow in deserts, and occasionally coastal dunes or salt marshes, where they are parasitic on the roots of various halophytic shrubs. *Cistanche* species are highly useful industrial cultivation plants resources for fixing sands and combating desertification.

Most species in *Cistanche* genus are traditionally used as nourishing and medicinal herbs, in particular, the species *C. deserticola* with the reputation of desert ginseng [5]. Several studies reported the chemical components and pharmacological effects of *Cistanche* species [6–8]. More than 100 compounds have been isolated from this genus, including phenylethanoid, glycosides, carbohydrates, lignans, iridoids, echinacoside, verbascoside, chlorogenic acid, acteoside, luteoloside, among others [9, 10]. These isolated compounds have exhibited interesting pharmacologic effects, such as neuroprotective, immunomodulatory, anti-senescence, anti-inflammatory, anti-osteoporosis, hepatoprotection, anti-oxidative, anti-bacterial, anti-tumor and glucose tolerance improving effects [10–12]. Driven by these medicinal properties and economic benefits, people overexploit and consume *Cistanche* species, giving to these species the importance highlighted in this work.

To date, four *Cistanche* species have been reported in China, namely *C. deserticola*, *C. salsa*, *C. sinensis* and *C. tubulosa* [13]. However, the classification of *Cistanche* remains controversial due to the following reasons: First, there are too few existing voucher specimens; second, the number of related literatures is scarce; third, the color of the flowers, in fresh and dried specimens, differ significantly becoming confusing the species classification based on flower color; fourth, the type of host plant is difficult to determine. Najibeh Ataeia et al. had reported molecular phylogeny analysis of a large-scale sampling of *Cistanche* species, indicating the presence of four clades based on their geographic distribution [14]. However, this study didn’t solve the problem of interspecies identification. In addition, the most economically important species, *C. deserticola* was not included. Therefore, we aimed to collect *Cistanche* species in China and determine their evolutionary status through phylogenetic analysis.

The chloroplast is an organelle peculiar to green plants, plays a crucial part in photosynthesis, and possess its own genome [15]. Chloroplast genomes are highly conserved, including genome size, structure, gene content, and organization [16, 17]. Consequently, the chloroplast genome is an excellent tool for phylogenetic analyses, genetic diversity evaluation, and molecular identification. In recent years, the complete chloroplast genome sequence has been successfully used as a plant super-barcode to distinguish closely related species in some taxa, such as *Paeonia L. *[18]. Whereas in other cases, chloroplast-derived DNA markers have been developed to authenticate medicinal plants as such, due to the lack of or reduced number of morphological based characters to differentiate among species, like the SNPs or insertion-deletion mutations (Indels) of the intergenic regions in the plastome of *Panax ginseng* species [19, 20].

During the process of our study, the plastic genome sequences of four *Cistanche* species were published. Severe gene loss and pseudogenization within *Cistanche* species plastomes were discussed, but only shown as supplementary material [1, 21]. In this work, for the first time, we used five full *Cistanche* species plastomes to: (1) explore structural variations within the *Cistanche* plastomes; (2) to illustrate the phenomena of gene loss and pseudogenization within *Cistanche* species; and (3) to develop taxa-specific molecular markers and DNA barcodes to distinguish between *Cistanche* species. The results here obtained have improved the understanding of relationships among *Cistanche* species, and will be invaluable for ensuring the effective and safe use of *Cistanche* species-based medicinal products.

## Results

### Sequencing and assembly of *Cistanche* plastomes

Illumina reads were mapped to the assembled plastomes, obtaining a mean coverage depth of 100–300 folds (Figure S1). The four plastomes displayed typical circular quadripartite structure, and showed a high degree of conservation in gene organization and structure (resumed in Figure S2). The quadripartite structure consisted in a Large Single-Copy (LSC) region (32,470–52,005 bp) and a Small Single-Copy (SSC) region (398–29,719 bp), separated by two Inverted Repeat (IR) regions (6,593–30,352 bp) (Table S1). The schematic presentation of the plastomes for *C. deserticola* (Figure S3), *C. salsa* (Figure S4), *C. tubulosa* (Figure S5) and *C. sinensis* (Figure S6) were shown. The sizes of *C. deserticola*, *C. salsa*, *C. sinensis* and *C. tubulosa* plastomes were 109, 454 bp, 111,690 bp, 111,500 bp and 75,735 bp long respectively (Table S1). The overall GC contents of *C. deserticola*, *C. salsa*, *C. sinensis* and *C. tubulosa* plastomes were 36.27%, 36.11%, 36.75% and 34.95%, respectively (Table S1). Generally, the GC content of SSC regions was lower than those of the LSC and IR regions, except for *C. tubulosa*. The gene contents of the five plastomes were listed in Table S1. The numbers of all genes, containing protein-coding genes (PCGs), putative pseudo genes, tRNA genes, and rRNA genes predicted as well as those possibly missing were counted (Table S1). Overall, 29–44 PCGs had been possibly lost. The numbers were similar to those for the PCGs identified in these plastomes, indicating the large scale of gene loss and gene erosion in *Cistanche* plastomes.

### Expansion and contraction of the SSC regions in the *Cistanche* plastomes

**Fig. 1:**
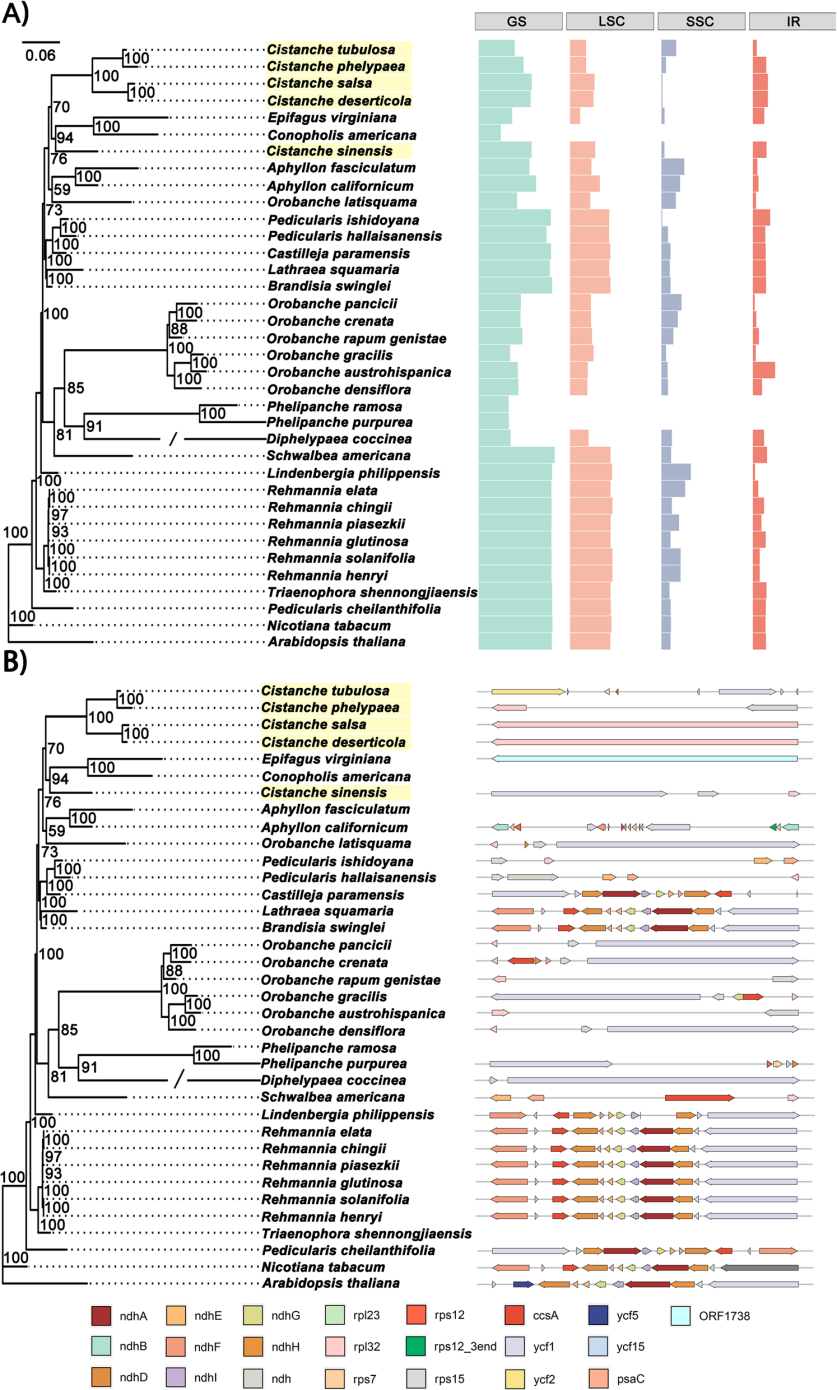
The variation of SSC regions in the plastomes for 36 *Orobanchaceae* species. **A**) Length of GS, LSC, SSC, and IR regions were plotted to the right of the ML tree. **B**) Protein coding gene in SSC region. The schematic representations of the SSC regions are plotted to the right of the ML tree

**Fig. 2:**
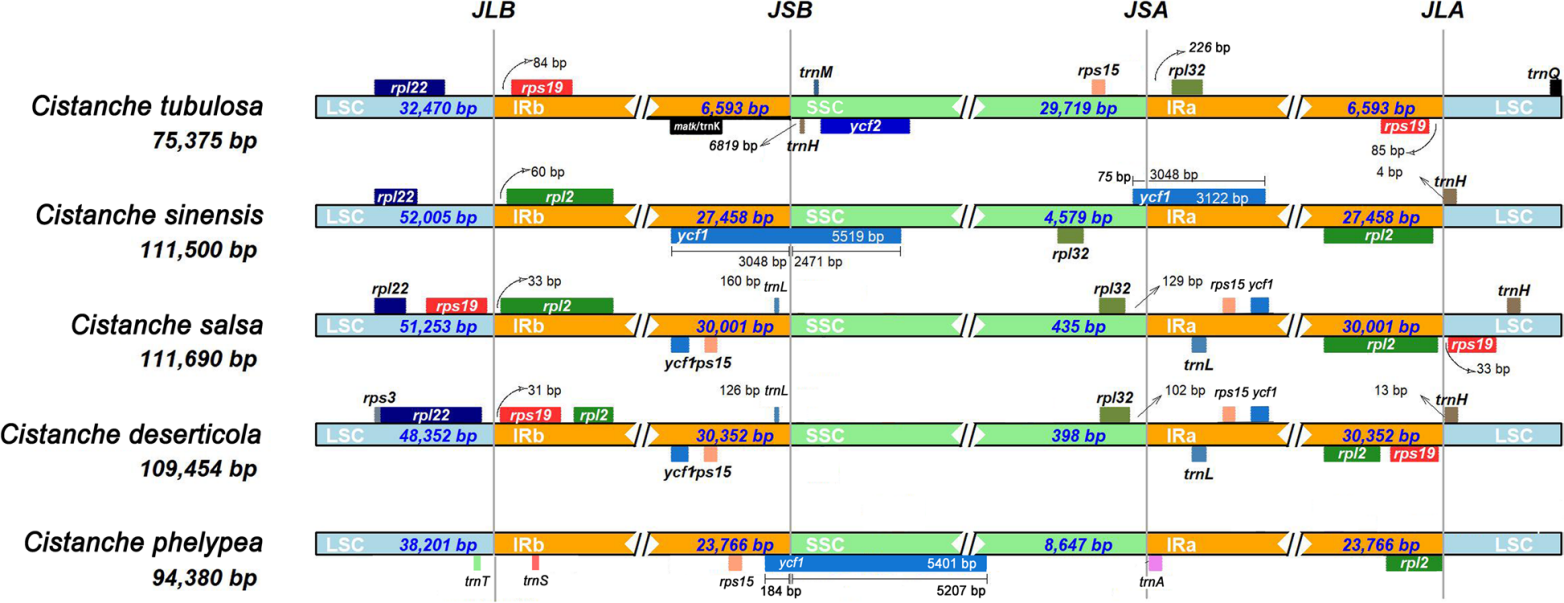
Analyses of expansion and contraction of inverted repeats of the five *Cistanche* species. The genes present at the top of track transcribe counterclockwise, whereas genes present below the track transcribe clockwise. Schematic representation of the boundary areas of LSC (light blue), IRa (yellow), SSC (green), and IRb (yellow) region for *Cistanche* species. The species names are shown to the left. The junction sites between LSC and IRb (*JLB*), IRb and SSC (*JSB*), SSC and IRa (*JSA*), IRa and LSC (*JLA*) are marked with the perpendicular dashed lines. The genes *rpl*22 (dark blue), *rps*19 (light red), *ycf*1 (blue), *ycf*2 (dark blue), *rps*15 (light orange), *rpl*32 (light green), *rpl*2 (green) are shown above the plastomes. The numbers above the gene features denote the distance between the gene borders, either the start or end of genes and the junction sites

The SSC regions of the *Cistanche* plastomes contracted significantly compared with the plastomes of other *Orobanchaceae* species, with the sizes ranging from only 27 bp to 61,091 bp long (Fig. 1 A, Table S2). In contrast, the IR regions expanded significantly, with the sizes ranging from 2, 318 bp to 45,796 bp, compared with the length of the IR regions of 15 other *Orobanchaceae* plastomes being larger than 25 kb (Table S1, Table S2). It is noticeable that the SSC regions in *C. deserticola* (398 bp) and *C. salsa* (435 bp) were extremely short, and only contained the *rpl*32 gene (Fig. 1B). The SSC regions of *C. tubulosa* was the longest and approximately 29,719 bp. Eight PCGs were located in the SSC regions of *C. tubulosa*, including *ycf*2, *ycf*15, *rps*7, *rps*12, *rpl*23, rpl32, *ycf*1 and *rps*15 (Table S4). These observations suggest that the SSC region of *Cistanche* being hotspots for gene loss, pseudogenization and rearrangement (Figure S10). The size variation in the SSC region mainly resulted from the gene loss and pseudogenization of *ndh*, *rps* and *ycf* gene (Table S3). Furthermore, the junctions of IR/LSC and IR/SSC were highly variable in the *Cistanche* plastomes due to the expansion/contraction of IRs (Fig. 2). The junction of the LSC/IRa (IRb) region of *C. deserticola* and *C. salsa* were highly conserved, with the *rpl*32 gene located at the SSC region (Table S4). But the distance of the *rpl*32 gene from the boundary varied (Fig. 2). Compared with *C. tubulosa*, the order of *rpl*32 and *rps*15 genes in *C. sals*a and *C. deserticola* was opposite. Another interesting observation was that the *ycf1* genes were located in both the IRb and SSC regions in *C. sinensis* and *C. phelypea* (Fig. 2). As shown in Figure S7, the plastomes were less conserved among these *Cistanche* species. A large number of genes were missing, including *rpo*A, *rpo*B, *rpo*C, *rpo*C2, *psa*B, *psa*D, *psa*I, *pet*A, *pet*B, *pet*D, *cem*A, etc. (Table 1 and Table S5). Moreover, compared with *C. deserticola*, some large inversions were identified in the plastomes of *C. salsa*, *C. sinensis*, *C. phelypaea* and *C. tubulosa* (Figure S8). While, five *Cistanche* species reveal varying degrees of genome loss when compared with the non-parasitic *Orobanchaceae* species *R. glutinosa* (Figure S9).

**Table 1:** Gene contents in the five plastomes of *Cistanche* species

Photosynthesis and energy production genes						Ribosomal RNA genes							Transfer RNA genes					
	Cd	Cs	Cc	Cp	Ct		Cd		Cs	Cc	Cp	Ct		Cd	Cs	Cc	Cp	Ct
*atp*A	Ψ	Ψ	Ψ	○	○	*rrn*16	•		•	•	•	•	*trn*A-UGC	•	•	•	•	○
*atp*B	Ψ	Ψ	Ψ	○	○	*rrn*23	•		•	•	•	•	*trn*C-GCA	•	•	•	•	•
*atp*E	Ψ	Ψ	Ψ	○	○	*rrn*4.5	•		•	•	•	•	*trn*D-GUC	•	•	•	•	•
*atp*F	Ψ	Ψ	○	○	○	*rrn*5	•		•	•	•	•	*trn*E-UUC	•	•	•	•	•
*atp*H	○	Ψ	○	○	○								*trn*F-GAA	•	•	•	•	•
*atp*I	○	Ψ	Ψ	○	○								*trn*G-GCC	•	•	•	•	•
*ndh*A	○	○	○	○	○								*trn*G-UCC	○	○	○	•	○
*ndh*B	Ψ	Ψ	Ψ	Ψ	Ψ								*trn*H-GUG	•	•	•	•	•
*ndh*C	○	○	○	○	○								*trn*I-CAU	○	○	○	•	○
*ndh*D	○	○	○	○	○	**RNA polymerase and intron maturase genes**							*trn*I-GAU	○	○	•	•	•
*ndh*E	○	○	○	○	○		Cd	Cs		Cc	Cp	Ct	*trn*K-UUU	•	•	•	•	•
*ndh*F	○	○	○	○	○	*mat*K	•	•		•	•	•	*trn*L-CAA	•	•	•	•	•
*ndh*G	○	○	○	○	○	*rpo*A	Ψ	Ψ		Ψ	○	○	*trn*L-UAA	•	•	•	•	•
*ndh*H	Ψ	Ψ	○	Ψ	○	*rpo*B	Ψ	Ψ		Ψ	Ψ	Ψ	*trn*L-UAG	•	•	•	•	•
*ndh*I	○	○	○	○	○	*rpo*C1	○	○		○	○	○	*trn*M-CAU	•	•	•	•	•
*ndh*J	○	○	Ψ	○	○	*rpo*C2	Ψ	Ψ		Ψ	Ψ	○	*trn*N-GUU	•	•	•	•	•
*ndh*K	○	○	Ψ	○	○								*trn*P-UGG	•	•	•	•	•
*pet*A	○	○	○	○	○								*trn*Q-UUG	•	•	•	•	•
*pet*B	○	○	Ψ	○	○								*trn*R-ACG	•	•	•	•	•
*pet*D	○	○	Ψ	○	○								*trn*R-UCU	•	•	•	•	○
*pet*G	Ψ	Ψ	Ψ	○	○	**Ribosomal protein and initiation factor genes**							*trn*S-CGA	•	•	○	○	○
*pet*L	○	○	○	○	○		Cd	Cs		Cc	Cp	Ct	*trn*S-GCU	•	•	•	•	•
*pet*N	○	○	○	○	○	*inf*A	•	•		•	•	•	*trn*S-GGA	•	•	•	•	•
*psa*A	Ψ	Ψ	Ψ	Ψ	Ψ	*rpl*14	•	•		•	•	•	*trn*S-UGA	•	•	•	•	•
*psa*B	Ψ	Ψ	Ψ	Ψ	Ψ	*rpl*16	•	•		•	•	•	*trn*T-GGU	•	•	•	•	•
*psa*C	○	○	○	○	○	*rpl*2	•	•		•	•	•	*trn*T-UGU	•	•	•	•	•
*psa*I	○	○	○	○	○	*rpl*20	•	•		•	•	•	*trn*V-GAC	•	•	•	•	•
*psa*J	○	○	○	•	○	*rpl*22	•	•		•	•	•	*trn*W-CCA	•	•	•	•	•
*psb*A	Ψ	Ψ	Ψ	Ψ	Ψ	*rpl*23	•	•		•	Ψ	•	*trn*Y-GUA	•	•	•	•	•
*psb*B	Ψ	Ψ	○	○	○	*rpl*32	•	•		•	○	•						
*psb*C	Ψ	Ψ	○	○	Ψ	*rpl*33	•	•		•	•	•						
*psb*D	Ψ	Ψ	○	○	○	*rpl*36	•	•		•	•	•						
*psb*E	Ψ	○	○	Ψ	Ψ	*rps*11	•	•		•	•	•						
*psb*F	Ψ	Ψ	○	○	○	*rps*12	•	•		•	•	•	**Other essential genes**					
*psb*H	○	○	○	○	○	*rps*14	•	•		•	•	•		Cd	Cs	Cc	Cp	Ct
*psb*I	○	○	Ψ	Ψ	○	*rps*15	•	•		•	•	•	*acc*D	•	•	•	•	•
*psb*J	○	○	○	Ψ	○	*rps*16	•	•		•	•	○	*ccs*A	○	○	○	Ψ	○
*psb*K	○	○	○	Ψ	○	*rps*18	•	•		•	•	•	*cem*A	○	○	○	○	○
*psb*L	Ψ	Ψ	○	○	○	*rps*19	•	•		•	•	•	*clp*P	•	•	•	•	•
*psb*M	Ψ	Ψ	Ψ	○	○	*rps*2	•	•		•	•	•	*ycf*1	•	•	•	Ψ	•
*psb*N	○	○	○	○	○	*rps*3	•	•		•	•	•	*ycf*15	•	•	Ψ	○	•
*psb*T	○	○	○	○	○	*rps*4	•	•		•	•	•	*ycf*2	•	•	•	•	•
*psb*Z	○	○	○	Ψ	○	*rps*7	•	•		•	•	•	*ycf*3	Ψ	Ψ	○	○	○
*rbc*L	Ψ	Ψ	Ψ	Ψ	Ψ	*rps*8	•	•		•	○	•	*ycf*4	○	○	○	Ψ	Ψ

*Cd C. deserticola*, *Cs C. salsa*, *Cc C. sinensis*, *Cp C. phelypaea*, *Ct*: *C. tubulosa*.•: genes present; Ψ: pseudogene; ○: genes missing

### Gene loss and pseudogenization in the *Cistanche* plastomes

**Fig. 3:**
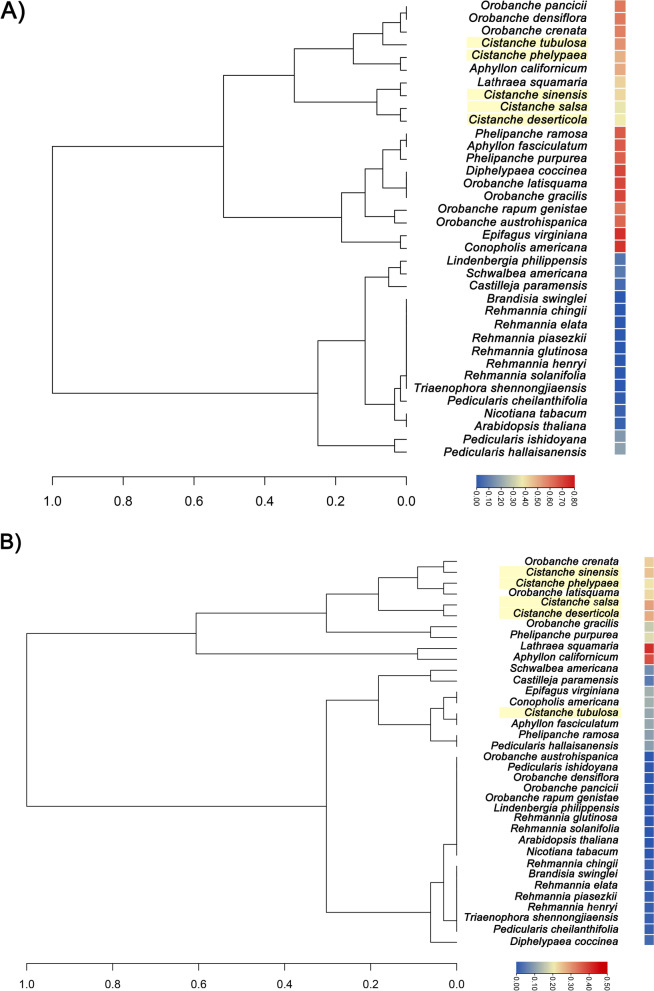
Inferred gene loss and pseudogenization in 36 *Orobanchaceae* species. Branch lengths of the tree are proportional to the maximum numbers of the lost genes or the pseudogenes. Heatmap is the ratio of lost genes to pseudogenes. Hierarchical clustering tree was conducted using dendro. **A**) Hierarchical clustering tree based on ratio of gene loss. **B**) Hierarchical clustering tree based on ration of pseudogenes

The SSC contraction and IR expansion were accompanied by gene loss and pseudogenization. We constructed a hierarchical clustering tree based on the ratio of gene loss and pseudogenes for 34 *Orobanchaceae* species, using *Arabidopsis thaliana* and *Nicotiana tabacum* as outgroups (Fig. 3). *Orobanchaceae* included nine genera, such as *Orobanche, Cistanche*, or *Phacellanthus*, among others. Gene loss was present in the plastomes of most *Orobanchaceae* species, except for *Rehmannia*, compared with *Arabidopsis thaliana* (Table S5). It was noteworthy that the plastomes of *Orobanchaceae* were characterized by a large percentage of gene losses, ranging from 41.25%—75%. The lost genes were mainly concentrated on the groups of photosynthesis and energy production genes, like *ndh* or *atp* genes. Among the *Cistanche* plastomes*, C. phelypaea* and *C. tubulosa* had the largest percentage of gene loss and *C. deserticola* and *C. salsa* had the smallest percentage of gene loss (Fig. 3A, Table 1 and Table S5).

All plastid genes of *Pedicularis ishidoyana*, *Orobanche austrohispanica*, *O. densiflora*, *O. pancicii*, *O. rapum genistae*, *Lindenbergia philippensis*, *Rehmannia glutinosa*, and *R. solanifolia* were functional. Pseudogenes were found most frequently in *Lathraea squamaria* and *Aphyllon californicum*, the percentages of which were 41.25% and 37.5%, respectively. Among the *Cistanche* plastomes*,* the proportion of pseudogenes ranged from 0.16 to 0.29, being ordered as *C. tubulosa* (0.16), *C. sinensis* (0.19), *C. deserticol*a (0.23), *C. phelypaea* (0.28), *C. salsa* (0.29) (Fig. 3B and Table S6). Most photosynthesis and energy production genes were pseudogenes in *Cistanche* species, including *ndh*, *psa*, *psb*, *rps*, *rpl* gene (Table S6).

### Nucleotide substitution rates of PCGs in *Cistanche* plastomes

**Fig. 4:**
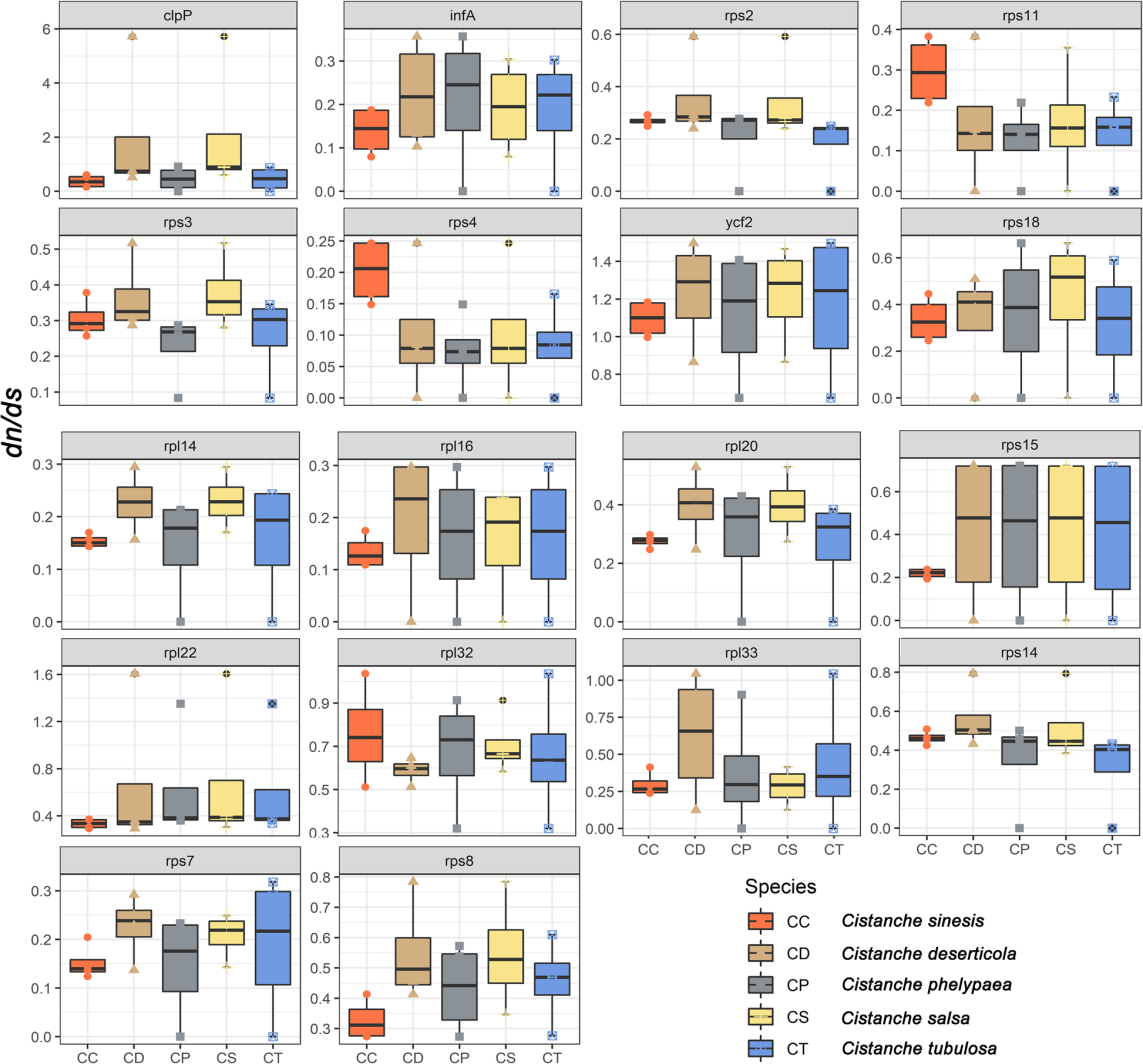
Boxplots of pairwise dN/dS values among each retained plastid genes within the five studied *Cistanche* species

The dN/dS ratio (*ω* = dN/dS) and aBSREL model analyses provide a comprehensive understanding of the evolution of genes by checking diversifying selection processes among related species. In general, the substitution rate for the *Cistanche* genus plastomes was low, indicating that plastid genes were highly conserved. However, we detected signals of positive selection in *ycf2* gene (*C. deserticola ω* = 1.24; *C. salsa ω* = 1.22; *C. tubulosa ω* = 1.17; *C. sinensis ω* = 1.10 and *C. phelypaea ω* = 1.12), and *chp*P and *rpl*22 gene within *C. phelypaea and C. salsa* (Fig. 4, Table 2). Other PCGs had mainly synonymous substitutions, with *rps*4 gene showing highest synonymous substitution rate (*ω* = 0.11) (Fig. 4).

**Table 2:** The positive selected genes in *Cistanche* branch

Genes	Branch	B	LRT	Test *p-value*	Uncorrected *p-value*	*ω* distribution over sites
*rpl*22	*C. phelypaea*	0.0035	21.7266	0.0000	0.0000	*ω*1 = *1.00 (99%)*
						*ω*2 = *10,000 (1.4%)*
*clp*P	*C. salsa*	0.0217	9.1315	0.0254	0.0036	*ω1* = *10,000,000,000 (100%)*

*B* Optimized branch length, *LRT* Likelihood ratio test statistic for selection, *Test p-value P-value* corrected for multiple testing, *Uncorrected p-value* Raw *p*-value without correction for multiple testing, ω *distribution over sites* Inferred ω estimates and respective proportion of sites

### Phylogenetic analysis and estimation of divergence time of *Cistanche* species

We inferred the species phylogenetic tree using the Maximum likelihood (ML) method with the shared PCGs among 36 *Orobanchaceae* species (Table S2, Figure S11), and then the tree was calibrated with fossil record data of *Arabidopsis thaliana*-*Nicotiana tabacum* with BEAST (Figure S12). The *Cistanche* species here tested shown a monophyletic origin as previously determined, with a low branch supporting value (BS: 66), being distributed in three main clades with *C. sinensis* as basal species/clade (Figure S11). The second clade contained *C. deserticola* and *C. salsa* (BS:100) (Figure S11)*,* both had similar plastome structure (Table S1). The third clade contained *C. tubulosa* and *C. phelypaea* (BS:100) (Figure S11), whose SSC region had similar length and composition (Table S1, Table S4). After this initial ML phylogenetic reconstruction, divergence time was established, with *Pedicularis cheilanthifolia* as root species of *Orobanchaceae* with divergence time estimated in 155 million years ago (Mya) (Figure S12). The monophyletic group of the *Cistanche* genus was diverged at about ~ 95.7 Mya, with *C. sinensis* splitting at ~ 77.3 Mya, and the remaining *Cistanch*e species clades separating ~ 45.2Mya. The split between *C. tubulosa* and *C. phelypaea* occurred approximately ~ 22.9 Mya, whereas *C. deserticola* and *C. salsa* were relatively young; their split was estimated to be ~ 12.3 Mya (Figure S12).

### Identification of highly variable intergenic regions in *Cistanche* species

**Fig. 5:**
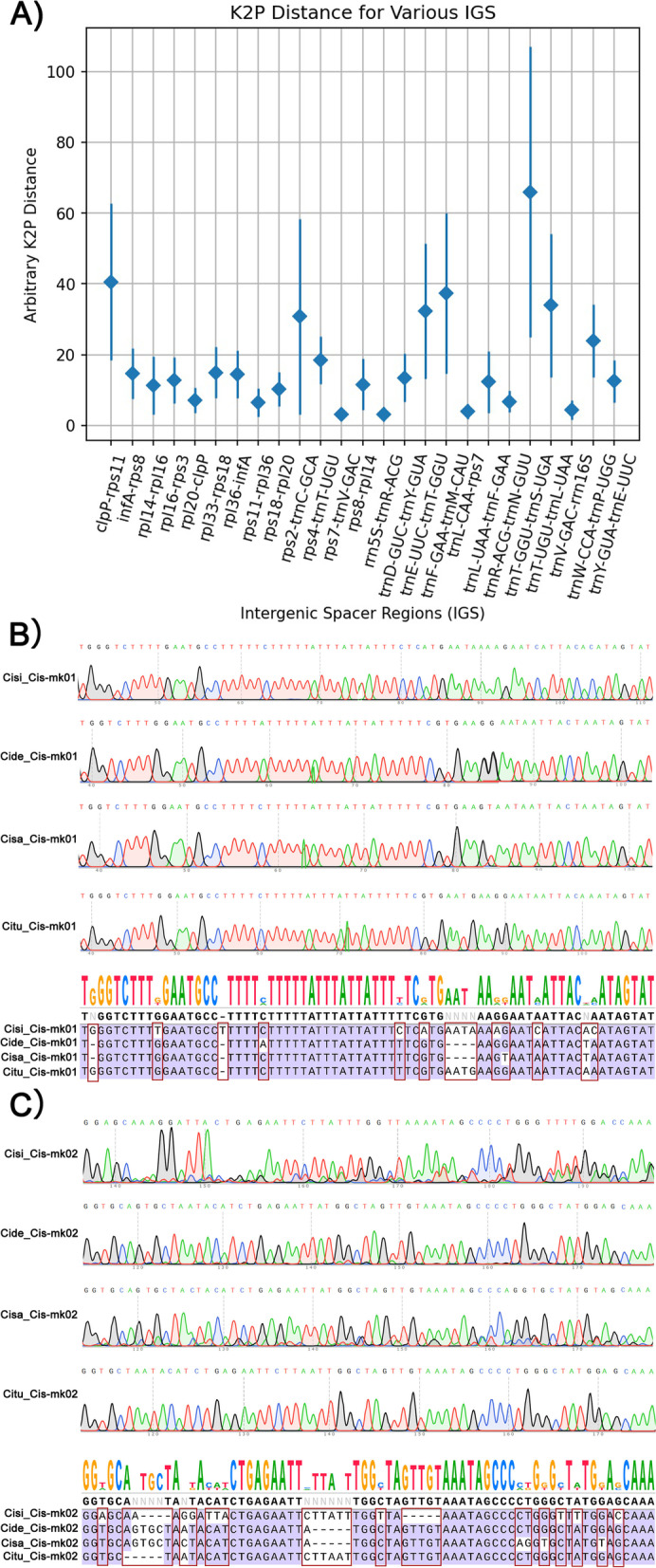
Identification of hyper variable intergenic spacer regions within *Cistanch*e species, and validation of the developed Cis-mk01 and Cis-mk02 DNA barcode markers. **A**) Comparison of the variability of IGS regions among the plastomes of *C. deserticola*, *C. salsa*, *C. tubulosa*, *C. sinensis* and *C. phelypaea*. The X-axis indicates the IGS regions; the Y-axis shows the range of K2p distances between different pairs of species, with the average K2p distance identified with a diamond. **B**) Sequencing chromatograms of the Cis-mk01 marker regions from *C. sinensis* (Cisi), *C. deserticola* (Cide), *C. salsa* (Cisa), and *C. tubulosa* (Citu), with consensus sequence and alignment. **C**) Sequencing chromatograms of the Cis-mk02 marker regions from *C. sinensis* (Cisi), *C. deserticola* (Cide), *C. salsa* (Cisa), and *C. tubulosa* (Citu), with consensus sequence and alignment

To identify the most suitable regions to develop *Cistanche* species-specific molecular markers Kimura two-parameter distances were determined in homologous intergenic regions (with dismat script on EMBOSS package; Fig. 5A). The most divergent intergenic regions among the five plastomes were the *clpP-rps11* (40.62), *rpl33-rps18* (14.97), *infA-rps8* (14.64), *rpl36-infA* (14.38), *rpl16-rps3* (12.81) and *rps18-rpl20* (10.18) (Fig. 5A).

### Development and validation of molecular markers for *Cistanche* species discrimination

We selected one of the hypervariable intergenic spacer regions (IGS) identified previously, *trn*R-ACG-*trn*N-GUU, to develop two DNA barcode markers named Cis-mk01 and Cis-mk02, respectively, with designed primers listed on Table S7. Markers were tested by PCR amplification on total DNAs from all four *Cistanche* samples (Figure S13). Sequence analysis of each PCR product allowed identifying that marker Cis-mk01 had nine specific SNP loci and three Indel loci (Fig. 5B) whereas marker Cis-mk02 had fourteen specific SNP loci and three Indel loci (Fig. 5 C). These SNP and indels allowed to differentiate successfully all the four *Cistanche* species.

### Specific DNA barcode maker design for *Cistanche* species

**Fig. 6:**
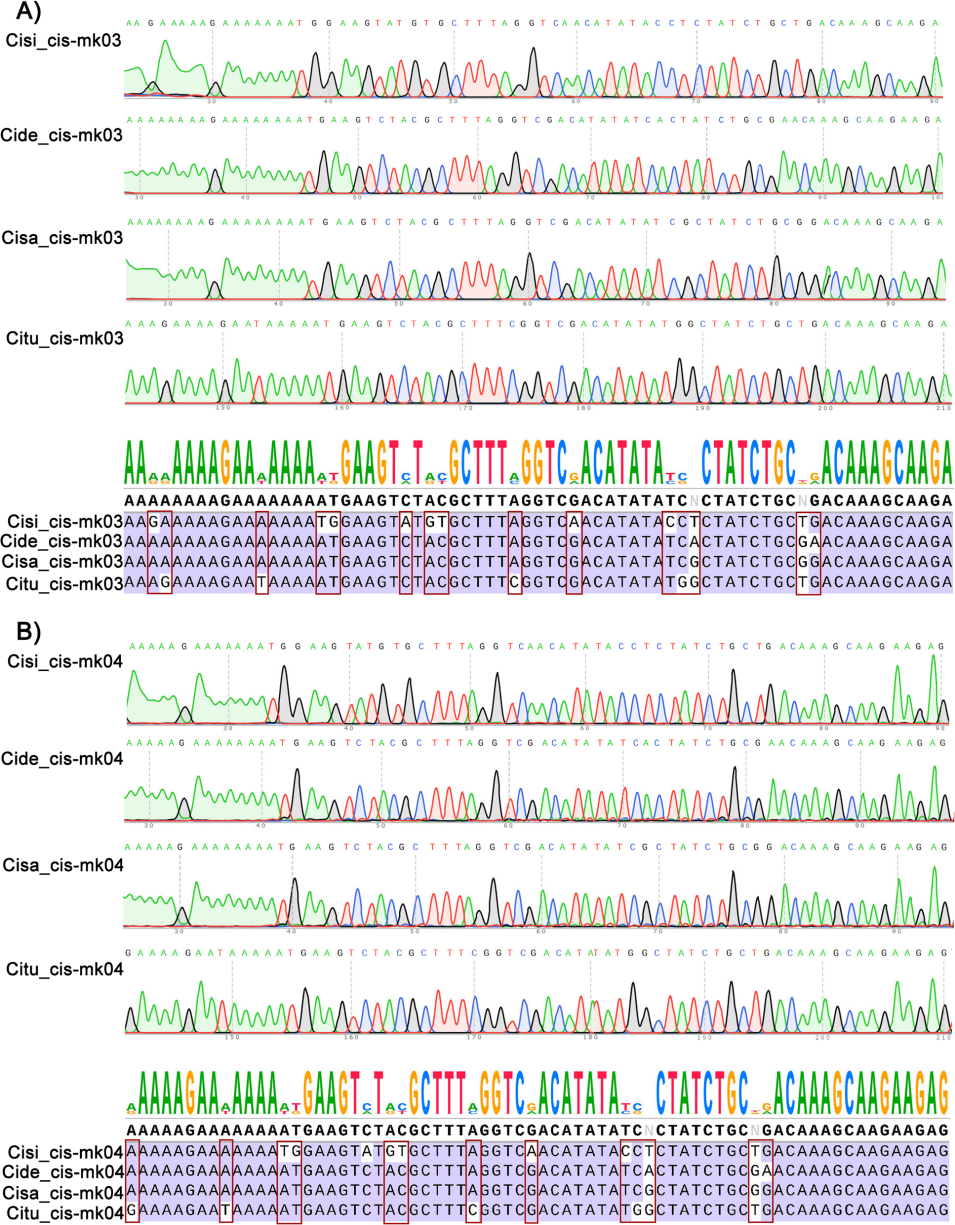
Validation of Cis-mk03 and Cis-mk04 developed to differentiate among *Cistanche *species. **A**) Sequencing chromatograms of Cis-mk03 regions from *C. sinensis* (Cisi), *C. deserticola* (Cide), *C. salsa* (Cisa), and *C. tubulosa* (Citu), with consensus sequence and alignment. **B**) Sequencing chromatograms of Cis-mk04 regions from *C. sinensis* (Cisi), *C. deserticola* (Cide), *C. salsa* (Cisa), and *C. tubulosa* (Citu), with consensus sequence and alignment

As explained in the introduction, highly variable regions in the plastome can be used for DNA barcode marker development. In this work, we used the EcoPrimer software to identify these hyper-variable regions as species-specific barcode and to design a pair of primers for each region for an accurate identification of each *Cistanche* species (Table S7). Eleven barcoding regions were verified (Fig. 6 and S14), of which Cis-mk03 showed 15 specific SNPs (Fig. 6A) and Cis-mk04 showed 14 specific SNPs (Fig. 6B) being able to differentiate successfully all the four *Cistanche* species used in this work. And the other nine of the eleven were shown in Figure S15.

## Discussion

### Large structural variations in *Cistanche* plastomes

Most plastomes of land plants are highly conserved in terms of genome structure, gene order and actual gene content, among other features [22, 23]. The plant plastomes are mainly between 115-165 kb long, have a typical quadripartite structure, and encode 105–135 genes, including 70–90 PCGs, eight rRNA genes and 30–40 tRNA genes, which are generally located on the LSC or SSC regions (about 15–20 genes are located in the IR region). In our study, we determined that the four *Cistanche* plastomes studied had a smaller size (less than 115 kb), and encoded less number of genes. These observations are consistent with the holoparasitic lifestyle of the *Cistanche* species. As they obtain the carbon, water, as well as other nutrients from their host’s roots or shoots, meaning that the photosynthetic ability in these plants is not needed, their plastomes are prone to gene reduction without affecting plants’ survivability [1, 24, 25]. Previous studies showed that the structural similarity of the five *Cistanche* species was relatively high [21]. However, our results shown that IR regions had shrunk and expanded to different degrees in the studied *Cistanche* species. Among them, the *C. deserticola* and the *C. salsa* had expanded dramatically (up to 30, 352 bp), while the *C. tubulosa* had contracted dramatically (down to 6, 593 bp). It is noteworthy that four rRNA genes (*rrn*16, *rrn*23, *rrn*4.5 and *rrn*5) were duplicated in the IRs in the *Cistanche* species studied except in *C. tubulosa*. In this species, these four rRNA genes are located as single copy, in the SSC region, explaining its relatively longer SSC and shorter IR (see Figure S5). Besides, as the most conservative region in plastomes, the sharp contraction of IRs indicated that *C. tubulosa* had gone through a much more intense gene loss, as reflected in its plastome size (*C. tubulosa*, 75, 375 bp), being the smallest of the five species under study. We speculated that this might be due to the tremendous difference in the growth environments of *Cistanche* species, leading to the significant different plastome structures among *Cistanche* species [26, 27].

### Extensive gene loss and pseudogenization in *Cistanche* plastomes

Compared to more than 7372 completely sequenced plastome of land plants, the number of fully sequenced plastomes of non-photosynthetic plants is very small (approximately 100 at the date of ending this study). To date, only a few plastomes of holoparasitic plants have been reported, such as some species from *Orobanchaceae* [24, 28], *Cuscutoideae* [29], and *Monotropa* [30] among others. In this study, we have sequenced the plastomes of four *Cistanche* species, a holoparasitic species from *Orobanchaceae*. The phylogenomic comparison with other non-parasitc plants will provide further insights on parasitic plastomes evolution.

Within the four new *Cistanche* plastomes, all the genes related to photosynthesis and energy production were pseudogenized or lost, in agreement with the holoparasitic and heterotrophic lifestyle of *Cistanche* genus. All *ndh* genes are either absent or pseudogenized, with the ratio of missing genes higher than pseudogenes. Chloroplast *ndh* genes encode subunits of the nicotinamide adenine dinucleotide-plastoquinone oxidoreductase complex, thought to reduce photo oxidative stress, which occurs when high light intensities exceed the capacity of the photosynthetic apparatus, resulting in the production of damaging reactive oxygen species [31]. *Cuscuta reflexa*, a holoparasitic plant within the *Convovulaceae*, appears to have an intact chloroplast genome, that also has lost all *ndh* genes [32], while genes *atp*, *pet*, *psa* and *psb* genes are either lost or pseudogenized. The ratio of pseudogenes is higher than those of lost genes in this species. Other parasitic species also have *atp*, *pet*, *psa* and *psb* genes as missing genes or as pseudogenes [33, 34]. Therefore, this phenomenon of gene loss and pseudogenization in *Cistanche* genus should be responsible for their simplified reduced plastome, common with other parasitic plant to adapt to their living style and environment.

### Consolidation of two species: *C. deserticola* and *C. salsa*

There has been a debate whether or not *C. deserticola* and *C. salsa* should be considered two distinct species [27, 35]. Previous work shows that *C. salsa* exhibited similar chemical composition and pharmacological activities to those of *C. deserticola*, and has been used as a local medicinal herb in Ningxia and Gansu province of China [36–38]. Likely suggesting that both species are really only one. Our findings revealed that *C. deserticola* and *C. salsa* had very similar plastome structures, lengths and composition (*C. deserticola* 109, 454 bp with 36.27% GC content and *C. salsa*, 111,690 bp with 36.11% GC content, see Table S1). With similar lengths of the typical four subregions (LSC, IRs, SSC), including the extremely short SSC region that seems be disappearing. The ML phylogenetic tree shown that *C. deserticola* and *C. salsa* gather together as a separate group from other *Cistanche* species (Figure S11). Moreover, the mean divergence time of these two sister species was estimated at 12.3 Mya. Furthermore, through field survey, we found that *C. salsa* has the same growing environment as *C. deserticola* but much larger reserves in nature. As a result, we propose that the two species should be considered one species. *C. salsa* might be utilized as *Cistanches* herb in Chinese Pharmacopoeia to solve the urgent problem of resource shortage.

## Conclusions

We presented a comparative analysis of five plastomes from *Cistanche* species, showing structure variation due to gene loss and pseudogenization, affecting mainly to photosynthetic genes. The genome sizes of these *Cistanche* species were smaller than that of other angiosperms. In particular, *C. deserticola* and *C. salsa* experienced significant SSC contraction and IR expansion, and *C. tubulosa* experienced SSC expansion and IR contraction. Phylogenetic relationships indicate that the *Cistanche* genus is a monophyletic group established around 95.7 Mya, with *C. deserticola* and *C.salsa* as two recognized species. Sequence variation among species allowed the development of two DNA barcode markers, Cis-mk01 and Cis-mk02, and eleven DNA barcode makers (Cis-mk03 to Cis-mk13) developed in a highly diverged intergenic region, that allow for the discrimination among *Cistanche* species from China. These results here obtained can improve our understanding on the classification system of *Cistanche*, plastome evolution, and the discrimination of medicinal products derived from *Cistanche* species.

## Methods

### Plant material and DNA extraction

**Fig. 7:**
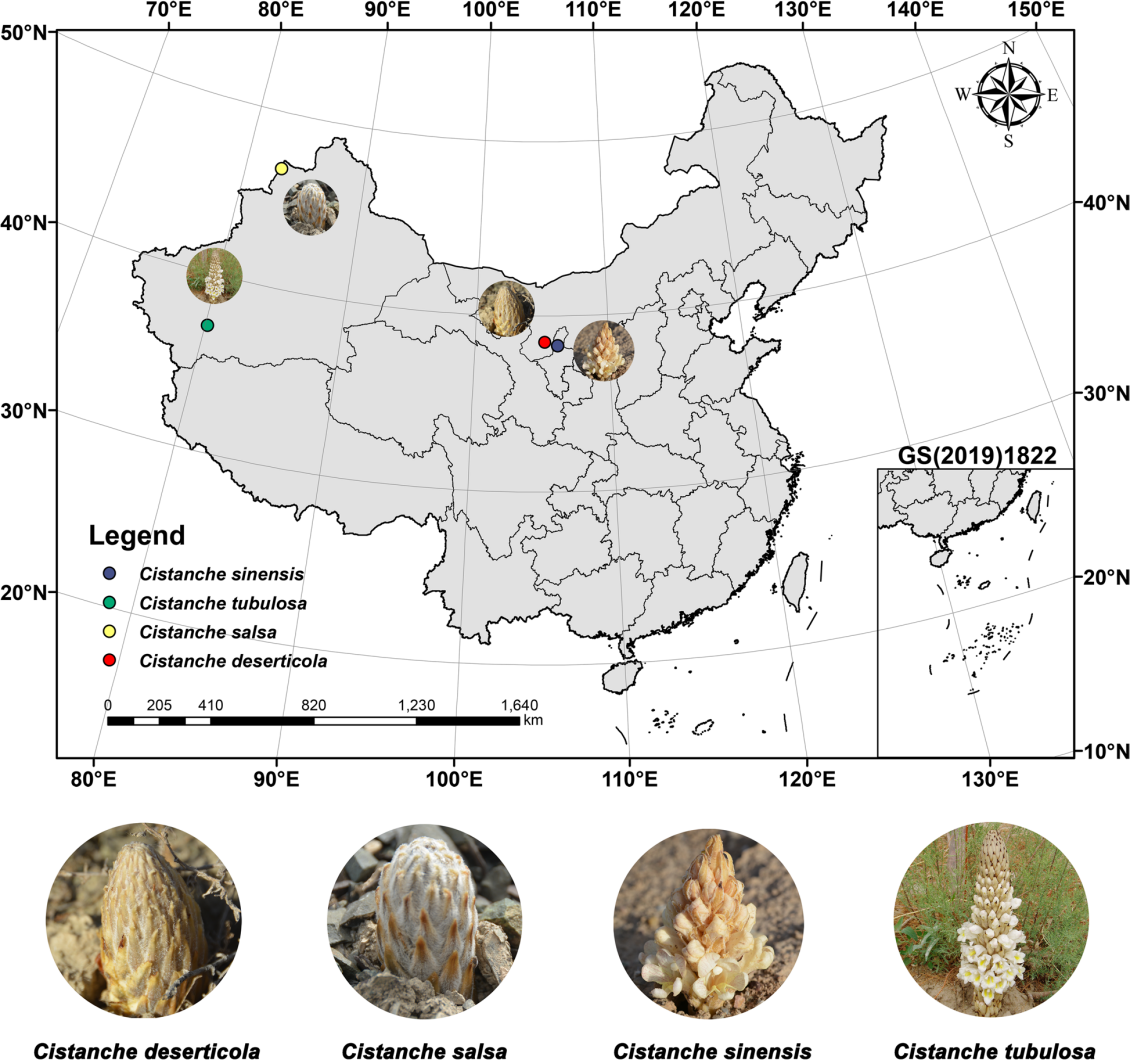
Geographical distribution and growing environment of four *Cistanche* species. Red dot: *Cistanche deserticola*, also known as ‘Rou Cong rong’ in Alxa League, Inner Mongolia Autonomous Region, China. Yellow dot: *Cistanche salsa*, also known as ‘Yan Sheng Rou Cong Rong’ in Tacheng City, Xinjiang Uygur Autonomous Region, China. Blue dot: *Cistanche sinensis*, also known as ‘Sha Cong Rong’ in Qingtongxia City, Ningxia Hui Autonomous Region, China. Green dot: *Cistanche tubulosa*, also known as ‘Guan Hua Rou Cong Rong’ in Hotan Prefecture, Xinjiang Uygur Autonomous Region, China

Fresh samples of *C. deserticola*, *C. salsa*, *C. sinensis*, and *C. tubulosa* were collected from the Alxa League (Inner Mongolia Autonomous Region), Tacheng City (Xinjiang Uygur Autonomous Region), Qingtongxia City (Ningxia Hui Autonomous Region), Hotan Prefecture (Xinjiang Uygur Autonomous Region), China, respectively (Fig. 7). Samples were taken from the *Cistanche* planting base without harming wild species, complying with local and national ethical requirements. The samples were identified by Professor Yulin Lin and stored at the Herbarium of the Chinese Academy of Medical Science & Peking Union Medicinal College (under specimens' registry numbers CMPB13484, CMPB13485, CMPB13486 and CMPB13487). Fresh samples were covered within tin foil, frozen with liquid nitrogen, and kept in -80 °C until use. A plant genomic DNA extraction kit (Tiangen Biotech, Beijing, China) was used for total DNA extraction. Total DNA quality was assessed by gel electrophoresis in 1% (w/v) agarose, and quantified in Qubit 3.0 (Life Technologies, Carlsbad, CA, USA) following manufacturer’s instructions.

### Genome sequencing, assembly and validation

About 500 ng DNA was utilized to build a paired-end library with an insert size of 500 bp following the manufacturer’s recommendations. The libraries were sequenced on an Illumina HiSeq 4000 platform (Illumina Inc., San Diego, CA, USA) following the manufacturer’s instructions. Consequently, a total of ~ 5G data for each species were obtained. All the plastomes of plants recorded in the GenBank were downloaded to build a local database. Clean paired-end reads were filtered against the local database by using BLASTn with a cutoff value of 1e-5. The filtered reads were used for downstream genome assembly with SPAdes (v. 3.10.1) [39]. To validate the correctness of the complete draft plastome, we mapped all raw reads to the draft plastome using Bowtie 2(v. 2.0.1) [40] and coverage graph was constructed by ggplot2 [41]. The dot plot of each plastome and themselves were constructed and visualized for evaluating the conserved quadripartite structure.

### Genome annotation, lost genes and pseudogenes identification

CpGAVAS2 web service [42] were used to annotate each of the four plastomes. Cutoffs for the E-values of BLASTn and BLASTx were set to 1E-10 to assign each gene, being edited manually with Apollo genome editor [43]. The circular gene maps were drawn in https://www.cloudtutu.com/. GC contents for each gene and plastome was determined with CGView Server [44]. The assembled plastomes have been deposited in GenBank with the accession numbers: MN614127 (*C. deserticola*), MN614128 (*C. salsa*), MN614129 (*C. sinensis*) and MN614130 (*C. tubulosa*). The plastome data of *Cistanche phelypaea* (NC_025642.1) was downloaded from NCBI and included in our downstream analysis.

Genes that were similar to known PCGs but were truncated or contained one or more frame shift mutations compared to the nonparasitic plant of *Orobanchaceae Rehmannia glutinosa* (NC_034308) were defined as pseudogenes [24]. Genes that missed compared to the nonparasitic plant of *Orobanchaceae R. glutinosa* (NC_034308) were defined as lost genes. The ratio of lost genes and pseudogenes was calculated in flowing formula: Ratio of lost genes (pseudogenes) = number of lost genes (pseudogenes)/ number of PCGs in *R. glutinosa*. Then we built the topology tree using R package ‘PD’ based on the ratio of lost genes and pseudogenes.

### Genome comparison

We conducted a comparative genome analysis for the *Cistanche* plastomes using software mVISTA [45] in the Shuffle-LAGAN mode. The annotated *Rehmannia glutinosa* [46] plastome was used as the reference in the analysis. Plastome genetic architecture of five *Cistanche* species in LSC/IRs and SSC/IRs borders were analyzed by IRscope [47]. Conserved sequences were identified between the five *Cistanche* plastomes by using BLASTN with an E-value cutoff of 1e-10. The homologous regions and gene annotations were visualized using a web-based genome synteny viewer GSV [48]. Gene rearrangements of the five *Cistanche* plastomes were analyzed using Mauve [49].

### Identification of hypervariable regions

To identify the most divergent regions, we wrote a custom script to extract the start and end of the IGS regions from the GenBank files for the five plastomes. A total of 25 IGSs shared by the five *Cistanche* plastomes were identified. The sequences were extracted and aligned using the ClustalW2 (v.2.0.12) program with options “-type = DNA –gapopen = 10 –gapext = 2” [50]. Pairwise distances were calculated using the K2p evolution model implemented in the distmat program from the EMBOSS package [51].

### Selective pressure analysis

Adaptive branch-site random effects likelihood (aBSREL) model [52] implemented in Hyphy [53] was used to determine the selective pressure analysis. We used the RevTrans v2.0 [54] to align the DNA sequences of 18 PCGs guided by their protein sequences with the option of CLUSTALW2. The significance analysis was performed using the likelihood ratio test (*p* ≤ 0.05) after correcting for multiple testing implemented in the program. We used the yn00programme in PAML v 4.9 [55] to calculate the nonsynonymous substitution rate (dN) and synonymous substitution rate(dS) for 18 PCGs with the F3X4 codon model. The ML tree inferred from shared PCGs was used as the input tree.

### Phylogenetic analyses

For phylogenetic analyses, the DNA sequences of shared plastome PCGs from 36 *Orobanchaceae* species including the *Cistanche* species of this work (Table S1) were aligned using ClustalW program [56]. The maximum likelihood (ML) tree was constructed with the software Randomized Axelerated Maximum Likelihood (RAxML) [57], using *Arabidopsis thaliana* and *Nicotiana tabacum* as outgroups. The detailed parameters were “raxmlHPC-PTHREADS-SSE3 -fa -N 1000 –m PROTGAMMACPREV/GTRGAMMA—× 551,314,260 -p 551,314,260 -o *Arabidopsis_thaliana*, *Nicotiana_tabacum* -T 20”. Bootstrap testing with 1,000 replications was used to assess the significant level of the phylogenetic tree, and the bootstrap values of each branch were displayed beyond each node.

### Molecular clock analyses

We used the software BEAST [58] for molecular clock analysis on the shared plastome PCGs alignment, using fossil information of *A. thaliana* and *N. tabacum*, and the cpREW amino acid substitution model for chloroplast DNA [59]. Phylogenetic inference following MCMC analysis with default settings was performed (20,000,000 generations, Yule speciation tree prior to the substitution rate, the trees sampled every 1,000 generations) under a strict clock approach. TRACER software was used to check the acceptability and convergence to the stationary distribution of trees [60], while TREEANNOTATOR software was used to generate the maximum clade credibility tree from the obtained trees after setting a burning-in of 10% [61]. The tree was visualized with FigTree (v. 1.4.3; http://tree.bio.ed.ac.uk/software/figtree/, October 2019).

### DNA barcode marker identification using EcoPrimer

EcoPrimer software was used to identify the DNA barcode maker based on the complete plastome sequences [62]. To achieve it, we download the *C. phelypaea* plastome and combined it with the four *Cistanche* plastomes sequences obtained in this study. The command "ecoPCRFormat.py-g-n Cistanche.Fo-t Taxonomy Cistanche.gb" was used to build the database. Subsequently, the command "ecoPrimer-d Cistanche.Fo-l 100-L 1000-e 0-t species > Cistanche.Po" was run on the constructed database to find specific primers for each of the DNA barcode markers.

### Identification and validation of molecular markers and ecoprimer for species discrimination

We used markers identified from the variable intergenic regions and ecoprimer to discriminate the four *Cistanche* species. Primers to discriminate between the four *Cistanche* species under study were designed on the variable intergenic regions using Snapgene 6.0 (Snapgene from Insightful Science, available at http://www.snapgene.com, last used on 2022), or were those selected with EcoPrimer software on the barcoding regions (Table S7 and S8). PCR amplifications were performed in a final volume of 20 μL with 10 μL 2 × Taq PCR Master Mix, 0. 5 μM of each primer, 5 μL template DNA, and 4 μL ddH_2_O following the manufacturer’s instructions (Mei5 Biotechnology, Co., Ltd). All amplifications were carried out in a Pro-Flex PCR system (Applied Biosystems, Waltham, MA, USA) under the following conditions: denaturation at 95 ℃ for 3 min, followed by 36 cycles of 94 ℃ for 25 s and 55℃ for 10 s, and 72 ℃ for 2 min as the final extension following the manufacturer’s instructions (Mei5 Biotechnology, Co., Ltd). PCR amplicons were visualized on 1% agarose gels, purified and then subjected to bidirectional Sanger sequencing on an ABI 3730 XL instrument (Applied Biosystems, USA) using the same set of primers used for PCR amplification with BigDye v3.1 chemistry (Applied Biosystems) following manufacturer’s instructions.

## Supplementary Information

**Additional file 1:** **Table S1.** Characteristics of the five* Cistanche *species plastomes. **Table S2**. Details of plastome or chloroplast genome sequences downloaded from NCBI used in this study. **Table S3. **Gene content in SSC region of *Orobanchaceae *species. **Table S4. **The position of genesin LSC, IR and SSC regions in five *Cistanche *species. **Table S5. **The list of lost genes in *Orobanchaceae *species. **Table S6. **The list of pseudogenes in *Orobanchaceae *species. **Table S7. **The thirteen pairs of primers for the ampilification of DNA barcode markers. **Fig. S1.** The coverage depth of the four *Cistanche *plastomes. The raw sequence reads were mapped to the reference plastome sequences. A) *C. deserticola*; B) *C. salsa*; C) *C. sinensis*; D) *C. tubulosa*. The X-axis shows the plastome positions. The Y-axis shows the depth. The Y-axis shows the coverage depth of the mapped reads. **Fig. S2.** The dot plots showing the self-2-selfalignment of the four *Cistanche *plastomes sequences. The plots were generated using Gepard. A) *C. deserticola*; B) *C. salsa*; C) *C. sinensis*; D) *C. tubulosa*. **Fig. S3. **A schematic map of the *Cistanche deserticola *plastome.The first circle shows the species name and specific information regarding the genome (length, GC content, and the number of genes) from the center going outward. The second circle shows the length of the corresponding single short copy (SSC), inverted repeat (IRa and IRb), and large single-copy (LSC) regions from the center going outward. The third circle shows the GC content. The outer circle shows the gene names and their optional codon usage bias in parentheses.The genes are colored based on their functional categories. Genes inside andoutside of the circle are transcribed in clockwise and counterclockwise directions, represented with arrows. **Fig. S4. **A schematic map of the *Cistanche salsa *plastome. The first circle shows the species name and specific information regarding the genome (length, GC content, and the number of genes)from the center going outward. The second circle shows the length of the corresponding single short copy (SSC), inverted repeat (IRa and IRb), and large single-copy (LSC) regions from the center going outward. The third circle shows the GC content. The outer circle shows the gene names and their optional codon usage bias in parentheses. The genes are colored based on their functional categories. Genes inside and outside of the circle are transcribed in clockwise and counterclockwise directions, represented with arrows. **Fig. S5. **Aschematic map of the *Cistanche tubulosa *plastome. The first circle showsthe species name and specific information regarding the genome (length, GCcontent, and the number of genes) from the center going outward. The second circle shows the length of the corresponding single short copy (SSC), invertedrepeat (IRa and IRb), and large single-copy (LSC) regions from the center going outward. The third circle shows the GC content. The outer circle shows the gene names and their optional codon usage bias in parentheses. The genes are coloredbased on their functional categories. Genes inside and outside of the circle are transcribed in clockwise and counterclockwise directions, represented with arrows. **Fig. S6. **A schematic map of the *Cistanche sinensis *plastome.The first circle shows the species name and specific information regarding thegenome (length, GC content, and the number of genes) from the center going outward. The second circle shows the length of the corresponding single shortcopy (SSC), inverted repeat (IRa and IRb), and large single-copy (LSC) regions from the center going outward. The third circle shows the GC content. The outer circle shows the gene names and their optional codon usage bias in parentheses.The genes are colored based on their functional categories. Genes inside andoutside of the circle are transcribed in clockwise and counterclockwise directions, represented with arrows. **Fig. S7. **Identity plot comparingthe plastid genomes of *C. deserticola*, *C. salsa*, *C. sinensis*,*C. tubulos*a and *C. phelypaea *using *Rehmannia glutinosa *as a reference sequence. The vertical scale indicates the percentage of identity(50% to 100%), using a 50% identity cutoff. The horizontal axis indicates the coordinates in the plastomes. Genome regions are color-coded as protein-coding, rRNA, tRNA, intron, and conserved non-coding sequences (CNS). **Fig. S8. **Synteny analyses of plastomes for five *Cistanche *species. Each horizontal black line represents a genome, with conserved regions connected with colored blocks. The plastome sequence of *C. deserticola *was used as the reference. **Fig.S9. **Synteny analyses of five *Cistanch*e plastomes compared with *Rehmannia glutinos*a. Each horizontal black line represents a genome, with conserved regions connected with colored block. The plastome of *R. glutinosa *was used as the reference. **Fig. S10. **Extent of the gene rearrangements of 5 *Cistanche *plastomes. Locally collinear blocks of the sequences are colour-coded and connected by lines. **Fig. S11. **Maximum likelihood (ML) Phylogenetic tree of 36 *Orobanchaceae *species. The *Cistanche *species are highlighted in blue. The bootstrap scores are shown on the corresponding branches. The detail information can be found in Table S2. **Fig. S12. **Maximum cladecredibility tree obtained from a molecules clock analysis using the BEAST software. The circles having different colors represent the genes positively selected in *Orobanchaceae*. The background of *Cistanche *is highlighted in light blue. The detail information can be found in Table S2. **Fig.S13. **The gel electrophoresis results of the PCR products amplified usingthe primers pairs list in Table S7 using DNA marker. Lane M was the marker of DL 2000. The lanes from left to right corresponded to products amplificated from the first individual of *C. deserticola *(Cide), *C. salsa *(Cisa),*C. tubulosa*(Citu), and *C. sinensis *(Cisi) by primer Cis-pp01 andCis-pp02, respectively. **Fig. S14. **The gel electrophoresis results of the PCR products amplified using the primers pairs list in Table S7 using DNAmarker. Lane M was the marker of DL 2000. The lanes from left to right corresponded to products amplificated from the first individual of *C.deserticola *(Cide), *C. salsa *(Cisa), *C. tubulosa *(Citu), andC. *sinensis *(Cisi) by primer Cis-pp03 to Cis-pp013, respectively. **Fig.S15. **The alignment of the sequencing chromatogram of the PCR products amplified using DNA marker (Cis-mk03 to Cis-mk13). SNP and Indel regions were highlighted with red squares. Cide: *C. deserticola*; Cisa: *C. salsa*;Citu: *C. tubulosa*; Cisi: *C.sinensis*. 

## Data Availability

The assembled plastid genomes of *C. deserticola*, *C. salsa*, *C. tubulosa* and *C. sinensis* were deposited in GenBank with the accession numbers MN614127, MN614128, MN614130 and MN614129.

## Abbreviations

CDS: Protein coding sequences
GC: Guanine-cytosine
IGS: Intergenic sequences
IR: Inverted repeat
IRa: Inverted repeat A
IRb: Inverted repeat B
IRs: Inverted repeat regions
kb: Kilobase
LSC: Large single copy
ML: Maximum likelihood
rRNAs: Ribosomal RNAs
SC: Single copy
SSC: Small single copy
tRNAs: Transfer RNAs
GS: Genome size
PCGs: Protein-coding genes

## References

[1] Li X, Zhang TC, Qin Q, Ren Z, Zhao J, Takahiro Y, Masami H, Crabbe M, Li J, Yang Z (2013). Complete chloroplast genome sequence of holoparasite *Cistanche deserticola* (*Orobanchaceae*) reveals gene loss and horizontal gene transfer from its host *Haloxylon ammodendron* (*Chenopodiaceae*). PLoS ONE.

[2] Westwood JH, Yoder JI, Timko MP, dePamphilis CW (2010). The evolution of parasitism in plants. Trends Plant Sci.

[3] Wickett NJ, Honaas LA, Wafula EK, Das M, Huang K, Wu BA, Landherr L, Timko MP, Yoder J, Westwood JH (2011). Transcriptomes of the Parasitic Plant Family *Orobanchaceae* Reveal Surprising Conservation of Chlorophyll Synthesis. Curr Biol.

[4] Thorogood CJ, Leon CJ, Lei D, Aldughayman M, Huang Lf, Hawkins JA (2021). Desert hyacinths: an obscure solution to a global problem?. Plants, People, Planet.

[5] Wang T, Zhang X, Xie W (2012). Cistanche deserticola Y. C. Ma, “Desert Ginseng”: A Review. Am J Chin Med.

[6] Bougandoura A, D’Abrosca B, Ameddah S, Scognamiglio M, Mekkiou R (2016). Chemical constituents and in vitro anti-inflammatory activity of Cistanche violacea Desf. (Orobanchaceae) extract. Fitoterapia.

[7] Tian S, Miao M, Bai M, Wei Z (2017). Phenylethanoid Glycosides of *Cistanche* on menopausal syndrome model in mice. Saudi Pharm J.

[8] Wang D, Wang H, Li G (2017). The antidepressant and cognitive improvement activities of the traditional Chinese herb *Cistanche*. Evid Based Complement Alternat Med.

[9] Zan K, Jiao XP, Guo LN, Zheng J, Shuang-Cheng MA (2016). HPLC specific chromatogram of Lamiophlomis Herba and its counterfeit and determination of four effective components. China J Chin Materia Med.

[10] Fu Z, Fan X, Wang X, Gao X (2018). *Cistanches* Herba: an overview of its chemistry, pharmacology, and pharmacokinetics property. J Ethnopharmacol.

[11] Wang N, Ji S, Zhang H, Mei S, Qiao L, Jin X (2017). Herba *Cistanches*: Anti-aging. Aging Dis.

[12] Morikawa T, Xie H, Pan Y, Ninomiya K, Muraoka O (2019). A Review of Biologically Active Natural Products from a Desert Plant *Cistanche tubulosa*. Chem Pharm Bull.

[13] Zhang Z, Tzvelev NN. Orobanchaceae. Flora of China. Beijing: Science press; 1998:18;229–43.

[14] Ataei N, Schneeweiss GM, Garcia MA, Krug M, Lehnert M, Valizadeh J, Quandt D (2020). A multilocus phylogeny of the non-photosynthetic parasitic plant *Cistanche* (*Orobanchaceae*) refutes current taxonomy and identifies four major morphologically distinct clades. Mol Phylogenet Evol.

[15] Brunkard JO, Runkel AM, Zambryski PC (2015). Chloroplasts extend stromules independently and in response to internal redox signals. Proc Natl Acad Sci USA.

[16] Tonti-Filippini J, Nevill PG, Dixon K, Small I (2017). What can we do with 1000 plastid genomes?. Plant J.

[17] Wicke S, Schneeweiss GM, dePamphilis CW, Muller KF, Quandt D (2011). The evolution of the plastid chromosome in land plants: gene content, gene order, gene function. Plant Mol Biol.

[18] Guo LL, Guo S, Xu J, He LX, Carlson JE, Hou XG (2020). Phylogenetic analysis based on chloroplast genome uncover evolutionary relationship of all the nine species and six cultivars of tree peony. Ind Crops Prod.

[19] Binh NV, Ngoc L, Espinosa WN, Hyun-Seung P, Nam-Hoon K, Woojong J, Junki L, Yang TJ (2020). Comprehensive comparative analysis of chloroplast genomes from seven *Panax* species and development of an authentication system based on species-unique single nucleotide polymorphism markers. J Ginseng Res.

[20] Kyunghee K, Sang-Choon L, Junki L, Oh LH, Jun JH, Nam-Hoon K, Hyun-Seung P, Yang TJ, Berthold H (2015). Comprehensive survey of genetic diversity in chloroplast genomes and 45S nrDNAs within *Panax ginseng* species. PLoS ONE.

[21] Liu XQ, Fu WR, Tang YW, Zhang WJ, Song ZP, Li LF, Yang J, Ma H, Yang JH, Zhou C (2020). Diverse trajectories of plastome degradation in holoparasitic *Cistanche* and genomic location of the lost plastid genes. J Exp Bot.

[22] Kato T, Kaneko T, Sato S, Nakamura Y, Tabata S (2000). Complete structure of the chloroplast genome of a legume. Lotus japonicus. DNA Res.

[23] De Las Rivas J (2002). Comparative analysis of chloroplast genomes: functional annotation, genome-based phylogeny, and deduced evolutionary patterns. Genome Res.

[24] Cusimano N, Wicke S (2016). Massive intracellular gene transfer during plastid genome reduction in nongreen *Orobanchaceae*. New Phytol.

[25] Park I, Song JH, Yang S, Kim WJ, Choi G, Moon BC (2019). *Cuscuta* species identification based on the morphology of reproductive organs and complete chloroplast genome sequences. Int J Mol Sci.

[26] Sun X, Li L, Pei J, Liu C, Huang L (2020). Metabolome and transcriptome profiling reveals quality variation and underlying regulation of three ecotypes for *Cistanche deserticola*. Plant Mol Biol.

[27] Wang Y, Zhang L, Du Z, Pei J, Huang L (2019). Chemical diversity and prediction of potential cultivation areas of *Cistanche* herbs. Sci Rep.

[28] Wicke S, Kai FM, Pamphilis C, Quandt D, Wickett NJ, Yan Z, Schneeweiss R (2013). Mechanisms of functional and physical genome reduction in photosynthetic and nonphotosynthetic parasitic plants of the broomrape family. Plant Cell.

[29] Funk HT, Berg S, Krupinska K, Maier UG, Krause K (2007). Complete DNA sequences of the plastid genomes of two parasitic flowering plant species, *Cuscuta reflexa* and *Cuscuta gronovii*. BMC Plant Biol.

[30] Gruzdev EV, Mardanov AV, Beletsky AV, Kochieva EZ, Ravin NV, Skryabin KG (2016). The complete chloroplast genome of parasitic flowering plant *Monotropa hypopitys*: extensive gene losses and size reduction. Mitochondrial DNA Part B-Resources.

[31] Quiles MJ (2010). Stimulation of chlororespiration by heat and high light intensity in oat plants. Plant, Cell Environ.

[32] Haberhausen G, Zetsche K (1994). Functional loss of all ndh genes in an otherwise relatively unaltered plastid genome of the holoparasitic flowering plant *Cuscuta reflexa*. Plant Mol Biol.

[33] Wickett NJ, Zhang Y, Hansen SK, Roper JM (2008). Functional gene losses occur with minimal size reduction in the plastid genome of the parasitic liverwort *Aneura** mirabilis*. Mol Biol Evol..

[34] Ravin N, Gruzdev E, Beletsky A, Mazur A, Prokhortchouk E, Filyushin M, Kochieva E, Kadnikov V, Mardanov A, Skryabin K (2016). The loss of photosynthetic pathways in the plastid and nuclear genomes of the non-photosynthetic mycoheterotrophic eudicot *Monotropa hypopitys*. BMC Plant Biol.

[35] Sun X, Pei J, Zhao L, Ahmad B, Huang LF (2021). Fighting climate change: soil bacteria communities and topography play a role in plant colonization of desert areas. Environ Microbiol.

[36] Jin XL, Zhang QR (1994). Recent progress in the study on chemical constituents of herba *Cistanche*. China J Chin Materia Med.

[37] Jiang Y, Tu PF (2009). Analysis of chemical constituents in *Cistanche* species. J Chromatogr A.

[38] Hai-Ning LV, Zeng KW, Song YL, Jiang Y, Tu PF (2016). Phytochemical and Pharmacological Overview of *Cistanche* Species. Recent Adv Polyphenol Res.

[39] Bankevich A, Nurk S, Antipov D, Gurevich AA, Dvorkin M, Kulikov AS, Lesin VM, Nikolenko SI, Pham S, Prjibelski AD (2012). SPAdes: a new genome assembly algorithm and its applications to single-cell sequencing. J Comput Biol.

[40] Langmead B, Salzberg SL (2012). Fast gapped-read alignment with Bowtie 2. Nat Methods.

[41] Wickham H. Getting started with ggplot2. ggplot2. New York: Springer; 2016:11–31.

[42] Shi LC, Chen HM, Jiang M, Wang LQ, Wu X, Huang LF, Liu C (2019). CPGAVAS2, an integrated plastome sequence annotator and analyzer. Nucleic Acids Res.

[43] Lewis SE, Searle SMJ, Harris N, Gibson M, Lyer V, Richter J, Wiel C, Bayraktaroglu L, Birney E, Crosby MA (2002). Apollo: a sequence annotation editor. Genome Biol.

[44] Grant JR, Stothard P (2008). The CGView server: a comparative genomics tool for circular genomes. Nucleic Acids Res.

[45] Frazer KA, Pachter L, Poliakov A, Rubin EM, Dubchak I (2004). VISTA: computational tools for comparative genomics. Nucleic Acids Res.

[46] Zeng SY, Zhou T, Han K, Yang YC, Zhao JH, Liu ZL (2017). The complete chloroplast genome sequences of six *Rehmannia* species. Genes.

[47] Amiryousefi A, Hyvonen J, Poczai P (2018). IRscope: an online program to visualize the junction sites of chloroplast genomes. Bioinformatics.

[48] Revanna KV, Chiu CC, Bierschank E, Dong Q (2011). GSV: a web-based genome synteny viewer for customized data. BMC Bioinformatics.

[49] Darling A, Mau B, Blattner FR, Perna A (2004). Mauve: multiple alignment of conserved genomic sequence with rearrangements. Genome Res.

[50] Thompson JD, Gibson TJ, Higgins DG. Multiple sequence alignment using clustalW and clustalX. Current Protocols in Bioinformatics. vol. unit 2.3. USA: Wiley; 2003:2.3.1-2.3.22. 10.1002/0471250953.bi0203s00 18792934

[51] Rice P, Longden I, Bleasby A (2000). EMBOSS: the European molecular biology open software suite. Trends Genet.

[52] Smith MD, Wertheim JO, Weaver S, Murrell B, Scheffler K, Pond SLK (2015). Less is more: an adaptive branch-site random effects model for efficient detection of episodic diversifying selection. Mol Biol Evol.

[53] Pond SLK, Frost SDW, Muse SV (2005). HyPhy: hypothesis testing using phylogenies. Bioinformatics.

[54] Wernersson R, Pedersen AG (2003). RevTrans: multiple alignment of coding DNA from aligned amino acid sequences. Nucleic Acids Res.

[55] Yang Z (2007). PAML 4: phylogenetic analysis by maximum likelihood. Mol Biol Evol.

[56] Thompson JD, Higgins DG, Gibson TJ (1994). CLUSTAL-W: improving the sensitivity of progressive multiple sequence alignment through sequence weighting, position-specific gap penalties and weight matrix choice. Nucleic Acids Res.

[57] Stamatakis A (2014). RAxML version 8: a tool for phylogenetic analysis and post-analysis of large phylogenies. Bioinformatics.

[58] Bouckaert R, Heled J, Kuhnert D, Vaughan T, Wu CH, Xie D, Suchard MA, Rambaut A, Drummond AJ (2014). BEAST 2: a software platform for bayesian evolutionary analysis. PLoS Comput Biol.

[59] Adachi J, Waddell PJ, Martin W, Hasegawa M (2000). Plastid genome phylogeny and a model of amino acid substitution for proteins encoded by chloroplast DNA. J Mol Evol.

[60] Rambaut A, Drummond AJ, Xie D, Baele G, Suchard MA (2018). Posterior summarization in bayesian phylogenetics using Tracer 1.7. Systematic Biol.

[61] Bouckaert R, Vaughan TG, Barido-Sottani J, Duchene S, Fourment M, Gavryushkina A, Heled J, Jones G, Kuhnert D, De Maio N (2019). BEAST 2.5: An advanced software platform for Bayesian evolutionary analysis. PLoS Computational Biol.

[62] Riaz T, Shehzad W, Viari A, Pompanon F, Taberlet P, Coissac E (2011). ecoPrimers: inference of new DNA barcode markers from whole genome sequence analysis. Nucleic Acids Res.

