# Supplementary material for: Structural mutations of small single copy (SSC) region in the plastid genomes of five Cistanche species and inter-species identification
Source: BMC Plant Biol. 2022 Aug 25;22:412. doi: 10.1186/s12870-022-03682-x (PMC9404617; doi:10.1186/s12870-022-03682-x)
Supplement: Supplementary file 1 — Additional file 1: Table S1. Characteristics of the five Cistanche species plastomes. Table S2. Details of plastome or chloroplast genome sequences downloaded from NCBI used in this study. Table S3. Gene content in SSC region of Orobanchaceae species. Table S4. The position of genesin LSC, IR and SSC regions in five Cistanche species. Table S5. The list of lost genes in Orobanchaceae species. Table S6. The list of pseudogenes in Orobanchaceae species. Table S7. The thirteen pairs of primers for the ampilification of DNA barcode markers. Fig. S1. The coverage depth of the four Cistanche plastomes. The raw sequence reads were mapped to the reference plastome sequences. A) C. deserticola; B) C. salsa; C) C. sinensis; D) C. tubulosa. The X-axis shows the plastome positions. The Y-axis shows the depth. The Y-axis shows the coverage depth of the mapped reads. Fig. S2. The dot plots showing the self-2-selfalignment of the four Cistanche plastomes sequences. The plots were generated using Gepard. A) C. deserticola; B) C. salsa; C) C. sinensis; D) C. tubulosa. Fig. S3. A schematic map of the Cistanche deserticola plastome.The first circle shows the species name and specific information regarding the genome (length, GC content, and the number of genes) from the center going outward. The second circle shows the length of the corresponding single short copy (SSC), inverted repeat (IRa and IRb), and large single-copy (LSC) regions from the center going outward. The third circle shows the GC content. The outer circle shows the gene names and their optional codon usage bias in parentheses.The genes are colored based on their functional categories. Genes inside andoutside of the circle are transcribed in clockwise and counterclockwise directions, represented with arrows. Fig. S4. A schematic map of the Cistanche salsa plastome. The first circle shows the species name and specific information regarding the genome (length, GC content, and the number of genes)from the center going out [file 12870_2022_3682_MOESM1_ESM.zip › Supplementary Information file 1.pdf]

Supplementary Information

Title: Structural mutations of small single copy (SSC) region in the plastid genomes of five *Cistanche* species and inter-species identification

Yujing Miao #, Haimei Chen #, Wanqi Xu, QiaoQiao Yang, Chang Liu*, & LinFang Huang *

Key lab of Chinese Medicine Resources Conservation, State Administration of Traditional Chinese Medicine of China, Institute of Medicinal Plant Development, Chinese Academy of Medical Sciences, Peking Union Medical College, Beijing 100193, China

* Corresponding author: cliu@implad.ac.cn; lfhuang@implad.ac.cn
# Yujing Miao and Haimei Chen contributed equally to this work.

Table S1. Characteristics of the five *Cistanche* species plastomes.

Species	*C. deserticola*	*C. salsa*	*C. sinensis*	*C. phelypaea*	*C. tubulosa*
**GenBank accession number**	MN614127 (This study)	MN61412 (This study)	MN14129 (This study)	NC_025642.1	MN614130 (This study)
**Total cpDNA size(bp)**	109,454	111,690	111,500	94,380	75,375
LSC(bp)	48,352	51,253	52,005	38,201	32,470
IR(bp)	30,352	30,001	27,458	23,766	6,593
SSC(bp)	398	435	4,579	8,647	29,719
**Total GC content(%)**	36.27	36.11	36.75	36.56	34.95
GC content of LSC (%)	32.82	32.56	32.73	32.77	32.87
GC content of IR (%)	39.11	39.23	41.41	40.57	33.22
GC content of SSC (%)	22.36	23.22	26.58	26.22	38.00
**Total number of genes**	80	81	77	73	64
Number of protein coding genes identified	28	28	27	24	27
Number of putative pseudo-genes	22	23	20	17	9
Number of lost protein coding genes	30	29	33	39	44
Number of tRNA genes identified	26	26	26	28	24
Number of lost tRNA genes	3	3	3	1	5
Number of rRNA genes identified	4(two copies)	4(two copies)	4(two copies)	4(two copies)	4(one copy)

Table S2. Details of plastome or chloroplast genome sequences downloaded from NCBI used in this study.

Organism Name	Family	RefSeq	Size(Kb)	LSC(bp)	SSC(bp)	IR(bp)	GC%	Type	Genes	CDS	tRNA
Aphyllon californicum	*Orobanchaceae*	NC_025651.1	120.84	62000	38126	10357	36.69	chloroplast	123	45	41
Aphyllon fasciculatum	*Orobanchaceae*	NC_039679.1	106.80	43970	47074	7876	34.66	chloroplast	91	34	36
Boulardia latisquama	*Orobanchaceae*	NC_025641.1	80.36	41343	29024	4997	35.75	plastid	93	29	31
Brandisia swinglei	*Orobanchaceae*	NC_042954.1	155.34	84650	17698	26498	38.07	chloroplast	133	87	37
Castilleja paramensis	*Orobanchaceae*	NC_031805.1	152.93	84105	17587	25617	38.19	plastid	133	84	37
Cistanche phelypaea	*Orobanchaceae*	NC_025642.1	94.38	32648	8082	26825	36.56	plastid	99	30	42
Conopholis americana	*Orobanchaceae*	NC_023131.1	45.67	-	-	-	33.94	plastid	56	21	19
Epifagus virginiana	*Orobanchaceae*	NC_001568.1	70.03	19799	4759	22735	36.00	chloroplast	71	25	23
Lathraea squamaria	*Orobanchaceae*	NC_027838.1	150.50	81981	16059	26232	38.13	chloroplast	130	50	37
Lindenbergia philippensis	*Orobanchaceae*	NC_022859.1	155.10	88034	61091	2989	37.79	chloroplast	137	85	37
Orobanche austrohispanica	*Orobanchaceae*	NC_031441.1	81.99	36198	11342	45796	35.09	chloroplast	59	29	26
Orobanche crenata	*Orobanchaceae*	NC_024845.1	87.53	43054	33119	5678	35.19	chloroplast	106	36	39
Orobanche densiflora	*Orobanchaceae*	NC_031442.1	83.02	35182	12086	17878	35.54	chloroplast	63	32	27
Orobanche gracilis	*Orobanchaceae*	NC_023464.1	65.53	48570	8189	4387	34.56	chloroplast	77	24	31
Orobanche pancicii	*Orobanchaceae*	NC_031443.1	88.53	42763	41126	2318	35.24	chloroplast	63	32	27
Orobanche rapum-genistae	*Orobanchaceae*	NC_031444.1	91.52	45127	23668	11361	35.50	chloroplast	62	31	27
Pedicularis cheilanthifolia	*Orobanchaceae*	NC_036010.1	155.16	85256	17971	25966	37.68	chloroplast	133	87	37
Pedicularis hallaisanensis	*Orobanchaceae*	NC_037433.1	143.47	81664	12203	24801	38.65	chloroplast	119	70	37
Pedicularis ishidoyana	*Orobanchaceae*	NC_029700.1	152.57	82018	27	35263	38.09	chloroplast	123	77	38
Phelipanche purpurea	*Orobanchaceae*	NC_023132.1	62.89	-	-	-	31.08	chloroplast	76	30	26
Phelipanche ramosa	*Orobanchaceae*	NC_023465.1	62.30	-	-	-	32.06	chloroplast	67	27	24
Phelypaea coccinea	*Orobanchaceae*	NC_043877.1	66.62	37964	20485	22294	31.74	chloroplast	70	26	32
Rehmannia chingii	*Orobanchaceae*	NC_033534.1	154.06	88982	20485	22294	37.97	chloroplast	133	87	37
Rehmannia elata	*Orobanchaceae*	NC_034312.1	153.77	84899	49011	9931	37.97	chloroplast	133	87	37
Rehmannia glutinosa	*Orobanchaceae*	NC_034308.1	153.62	84778	17580	25632	37.95	chloroplast	133	88	37
Rehmannia henryi	*Orobanchaceae*	NC_034309.1	153.89	88935	39061	12947	37.95	chloroplast	133	87	37
Rehmannia piasezkii	*Orobanchaceae*	NC_034311.1	153.93	85026	35713	16593	37.94	chloroplast	133	87	37
Rehmannia solanifolia	*Orobanchaceae*	NC_034310.1	153.99	88935	39158	12948	37.94	chloroplast	133	88	37

Schwalbea americana	*Orobanchaceae*	NC_023115.1	160.91	84756	18900	28627	38.08	chloroplast	129	82	37
Triaenophora shennongjiaensis	*Orobanchaceae*	NC_039781.1	155.32	84901	15450	27484	37.75	chloroplast	133	87	37
Arabidopsis thaliana	*Brassicaceae*	NC_000932.1	154.48	84170	17780	26264	36.29	chloroplast	129	85	37
Nicotiana tabacum	*Solanaceae*	NC_001879.2	155.94	86686	18571	25343	37.85	plastid	144	98	37

Table S3. Gene content in SSC region of *Orobanchaceae* species.

Species	Protein-coding gene
Cistanche tubulosa	*ycf*1,*ycf*15,*ycf7*,*rpl*23,*ycf*1,*rps*15,*rpl*32
Cistanche phelypaea	*rpl*32,*rps*15
Cistanche salsa	*rpl*32
Cistanche deserticola	*rpl*32
Epifagus virginiana	*ORF*1738
Cistanche sinensis	*ycf*1,*rps*15,*rpl*32
Pedicularis ishidoyana	*rps*15,*rpl*32,*ndh*E,*psa*C
Pedicularis hallaisanensis	*rps*15,*ndh*,*ndh*E,*psa*C,*rpl*32
Castilleja paramensis	*ycf*1,*rps*15,*ndh*H,*ndh*A,*ndh*I,*ndh*G,*ndh*E,*psa*C,*ndh*D,*ccs*A,*rpl*32,*ndh*F
Lathraea squamaria	*ndh*F,*rpl*32,*ccs*A,*ndh*D,*psa*C,*ndh*E,*ndh*G,*ndh*I,*ndh*A,*ndh*H,*rps*15,*ycf*1
Brandisia swinglei	*ndh*F,*rpl*32,*ccs*A,*ndh*D,*psa*C,*ndh*E,*ndh*G,*ndh*I,*ndh*A,*ndh*H,*rps*15,*ycf*1
Orobanche rapum genistae	*rpl*32,*rps*15
Orobanche austrohispanica	*rpl*32,*rps*15
Rehmannia chingii	*ndh*F,*rpl*32,*ccs*A,*ndh*D,*psa*C,*ndh*E,*ndh*G,*ndh*I,*ndh*A,*ndh*H,*rps*15,*ycf*1
Rehmannia glutinosa	*ndh*F,*rpl*32,*ccs*A,*ndh*D,*psa*C,*ndh*E,*ndh*G,*ndh*I,*ndh*A,*ndh*H,*rps*15,*ycf*1
Triaenophora shennongjiaensis	*ndh*F,*rpl*32,*ccs*A,*ndh*D,*psa*C,*ndh*E,*ndh*G,*ndh*I,*ndh*A,*ndh*H,*rps*15,*ycf*1
Pedicularis cheilanthifolia	*ycf*1,*rps*15,*ndh*H,*ndh*A,*ndh*I,*ndh*G,*ndh*E,*psa*C,*ndh*D,*ccs*A,*rpl*32,*ndh*F
Arabidopsis thaliana	*rpl*32,*ycf*5,*ndh*D,*psa*C,*ndh*E,*ndh*G,*ndh*I,*ndh*A,*ndh*H,*rps*15,*ycf*1
Nicotiana tabacum	*ndh*F,*rpl*32,*ccs*A,*ndh*D,*psa*C,*ndh*E,*ndh*G,*ndh*I,*ndh*A,*ndh*H,*rps*15,*ycf*1

Table S4. The position of genes in LSC, IR and SSC regions in five *Cistanche* species.

Species	LSC	IR	SSC
C. deserticola	*trn*H-GUG, *Ψpsb*A, *trn*K-UUU, *mat*K, *rps*16, *trn*Q-UUG, *trn*S-GCU, *trn*S-CGA, *trn*R-UCU, *Ψatp*A, *Ψatp*F, *rps*2, *rpo*C2, *trn*C-GCA, *psb*M, *trn*D-GUC, *trn*Y-GUA, *rn*E-UUC, *trn*T-GGU, *Ψpsd*D, *Ψpsd*C, *trn*S-UGA, *trn*G-GCC, *trn*M-CAU, *rps*14, *Ψpsa*B, *Ψpsa*A, *Ψycf*3, *trn*S-GGA, *rps*4, *trn*T-UGU, *trn*L-UAA, *trn*F-GAA, *trn*M-CAU, *Ψatp*E, *ΨatpB*, *Ψrbc*L, *acc*D, *Ψpsb*L, *Ψpsb*F, *Ψpsb*E, *Ψpet*G, *trn*W-CCA, *trn*P-UGG, *rpl*33, *rps*18, *rpl*20, *rps*12, *clp*P, *rps*11, *Ψpsb*B, *Ψrpo*A, *rpl*36, *inf*A, *rps*8, *rpl*14, *rpl*16, *rps*3, *rpl*22	*rps*19, *rpl*2, *trn*M-CAU, *ycf*2, *ycf*15, *trnL*-CAA, *Ψndh*B, *rps*7, *rps*12, *trnV*-GAC, *rrn*16S, *trn*E-UUC, *trnA*-UGC, *rrn*23S, *rpl*23, *rrn*4.5S, *rrn*5S, *trn*R-ACG, *trn*N-GUU, *ycf*1, *rps*15, *Ψndh*H, *trn*L-UAG	*rpl*32
C. salsa	*trn*H-GUG, *Ψpsb*A, *trn*K-UUU, *mat*K, *rps*16, *trn*Q-UUG, *trn*S-GCU, *trn*S-CGA, *trn*R-UCU, *Ψatp*A, *Ψatp*F, *Ψatp*H, *Ψatp*I, *rps*2, *rpo*C2, *trn*C-GCA, *psb*M, *trn*D-GUC, *trn*Y-GUA, *trn*E-UUC, *trn*T-GGU, *Ψpsd*D, *Ψpsd*C, *trn*S-UGA, *trn*G-GCC, *trn*M-CAU, *rps*14, *Ψpsa*B, *Ψpsa*A, *Ψycf*3, *trn*S-GGA, *rps*4, *trn*T-UGU, *trn*L-UAA, *trn*F-GAA, *trn*M-CAU, *Ψatp*E, *Ψatp*B, *Ψrbc*L, *acc*D, *psa*I, *Ψpsb*L, *rps*14, *Ψpsb*F, *Ψpet*G, *trn*W-CCA, *trn*P-UGG, *rpl*33, *rps*18, *rpl*20, *rps*12, *clp*P, *rps*11, *Ψpsb*B, *Ψrpo*A, *rpl*36, *inf*A, *rps*8, *rpl*14, *rpl*16, *rps*3, *rpl*22	*rps*19, *rpl*2, *trn*M-CAU, *ycf*2, *ycf*15, *trn*L-CA*A*, *Ψndh*B, *rps*7, *rps*12, *trn*V-GAC, *rrn*16S, *trn*E-UUC, *trn*A-UGC, *rrn*23S, *rpl*23, *rrn*4.5S, *rrn*5S, *trn*R-ACG, *trn*N-GUU, *ycf*1, *rps*15, *Ψndh*H, *trn*L-UAG	*rpl*32
C. sinensis	*trn*H-GUG, *Ψpsb*A, *trn*K-UUU, *mat*K, *rps*16, *trn*Q-UUG, *trn*S-GCU, *psb*I, *trn*R-UCU, *Ψatp*A, *Ψatp*I, *rps*2, *rpo*C2, *Ψrpo*B, *trn*C-GCA, *psb*M, *trn*D-GUC, *trn*Y-GUA, *rn*E-UUC, *trn*T-GGU,	*rpl*2, *rpl*23, *trn*M-CAU, *ycf*2, *ycf*15, *trn*L-CAA, *Ψndh*B, *rps*7, *trn*V-GAC, *rrn*16S, *trn*I-GAU, *trn*A-UGC, *rrn*23S, *rrn*4.5S, *rrn*5S, *trn*R-ACG, *trn*N-GUU, *ycf*1	*ycf*1, *rps*15, *rpl*32, *trn*L-UAG

	*trn*S-UGA, *trn*G-GCC, *trn*M-CAU, *rps*14, *Ψpsa*B, *Ψpsa*A, *trn*S-GGA, *rps*4, *trn*T-UGU, *trn*L-UAA, *trn*F-GAA, *Ψndh*J, *Ψndh*K, *trn*M-CAU, *Ψatp*E, *Ψatp*B, *Ψ*rbcL, *acc*D, *Ψpet*G, *trn*W-CCA, *trn*P-UGG, *rpl*33, *rps*18, *rpl*20, *clp*P, *Ψpet*B, *Ψpet*D, *Ψrpo*A, *rps*11, *rpl*36, *inf*A, *rps*8, *rpl*14, *rpl*16, *rps*3, *rpl*22, *rps*19		
C. phelypaea	*rps*16, *trn*Q-UUG, *Ψpsb*K, *Ψpsb*I, *trn*S-GCU, *trn*G-GCC, *trn*R-UCU, *rps*2, *Ψrpo*C2, *Ψrpo*B, *trn*C-GCA, *trn*D-GUC, *trn*Y-GUA, *trn*E-UUC, *trn*T-GGU, *trn*S-UGA, *Ψpsb*Z, *trn*G-UCC, *trn*M-CAU, *rps*14, *Ψpsa*B, *Ψpsa*A, *trn*S-GGA, *rps*4, *trn*T-UGU, *trn*L-UAA, *trn*F-GAA, *trn*M-CAU, *Ψrbc*L, *acc*D, *Ψycf*4, *Ψpsb*J, *Ψpsb*E, *trn*W-CCA, *trn*P-UGG, *psa*J, *rpl*33, *rps*18, *rpl*20, *rps*12, *clp*P, *rps*11, *rpl*36, *inf*A, *rps*8, *rpl*14, *rpl*16, *rps*3, *rpl*22, *rps*19, *rpl*2	*Ψrpl*2, *Ψrpl*23, *trn*K-UUU, *mat*K, *Ψpsb*A, *trn*H-GUG, *trn*M-CAU, *ycf*2, *trn*L-CAA, *Ψndh*B, *rps*7, *rps*12, *trn*V-GAC, *rrn*16S, *trn*I-GAU, *trn*A-UGC, *rrn*23S, *rrn*4.5S, *rrn*5S, *trn*R-ACG, *trn*N-GUU	*Ψycf*1, *rpl*32, *trn*L-UAG, *Ψccs*A, *Ψndh*H, *rps*15
C. tubulosa	*trn*Q-UUG, *trn*S-GCU, *rps*2, *Ψrpo*B, *trn*C-GCA, *trn*D-GUC, *trn*Y-GUA, *trn*E-UUC, *trn*T-GGU, *Ψpsb*C, *trn*S-UGA, *trn*G-GCC, *trn*M-CAU, *rps*14, *Ψpsa*B, *Ψpsa*A, *trn*S-GGA, *rps*4, *trn*T-UGU, *trnL*-UAA, *trn*F-GAA, *trn*M-CAU, *Ψrbc*L, *acc*D, *Ψtcf*4, *Ψpsb*E, *trn*W-CCA, *trn*P-UGG, *rpl*33, *rps*18, *rpl*20, *rps*12, *clp*P, *rps*11, *rpl*36, *inf*A, *rps*8, *rpl*14, *rpl*16, *rps*3, *rpl*22	*rps*19, *rpl*2, *trn*K-UUU, *mat*K, *Ψpsb*A	*trn*H-GUG, *trn*M-CAU, *ycf*2, *ycf*15, *trn*L-CAA, *Ψndh*B, *rps*7, *rps*12, *trn*V-GAC, *rrn*16S, *trn*I-GAU, *rrn*23S, *rpl*23, *rrn*5S, *rrn*4.5S, *trn*R-ACG, *trn*N-GUU, *ycf*1, *rps*15, *trn*L-UAG, *rpl*32

Table S5. The list of lost genes in *Orobanchaceae* species. (Please refer to the associated excel file)

Table S6. The list of pseudogenes in *Orobanchaceae* species. (Please refer to the associated excel file)

Table S7. The thirteen pairs of primers for the ampilification of DNA barcode markers.

Marker ID	Primer pair ID	Forward primer sequence 5'-3'	Reverse primer sequence 5'- 3'
Cis-mk01	Cis-pp01	GGGAAGTACCTTTCCCTCTG	AAGAAGAGTTTCGGGGCGTA
Cis-mk02	Cis-pp02	TTTTTTTGTTTTACGCCCTT	AATCCTTTGCTCCATAGCCC
Cis-mk03	Cis-pp03	ACAAACATATAGATAGGTACTTATT	GTTTGAATTTCTTCGTGGTAGTACC
Cis-mk04	Cis-pp04	ACATATAGATAGGTACTTATTATTC	CGCGGATTTGATCAATTACTCTTCT
Cis-mk05	Cis-pp05	ATCGTCCGCGGATTTGATCAATTAC	CAAAAACAAACATATAGATAGGTAC
Cis-mk06	Cis-pp06	AACAAACATATAGATAGGTACTTAT	GTCCGCGGATTTGATCAATTACTCT
Cis-mk07	Cis-pp07	AACAAACATATAGATAGGTACTTAT	AATTTACAAATAGGATAACACGCTC
Cis-mk08	Cis-pp08	AACAAACATATAGATAGGTACTTAT	GAATTTCTTCGTGGTAGTACCCTCA
Cis-mk09	Cis-pp09	ACAAACATATAGATAGGTACTTATT	ATCGTCCGCGGATTTGATCAATTAC
Cis-mk10	Cis-pp10	ACATATAGATAGGTACTTATTATTC	CCTCATAAGATCGTCCGCGGATTTG
Cis-mk11	Cis-pp11	CAAAAACAAACATATAGATAGGTAC	GATCAATTACTCTTCTTGCTTTGTT
Cis-mk12	Cis-pp12	CAAACATATAGATAGGTACTTATTA	GAATTTCTTCGTGGTAGTACCCTCA
Cis-mk13	Cis-pp13	ATTTACAAATAGGATAACACGCTCT	CAAAAACAAACATATAGATAGGTAC

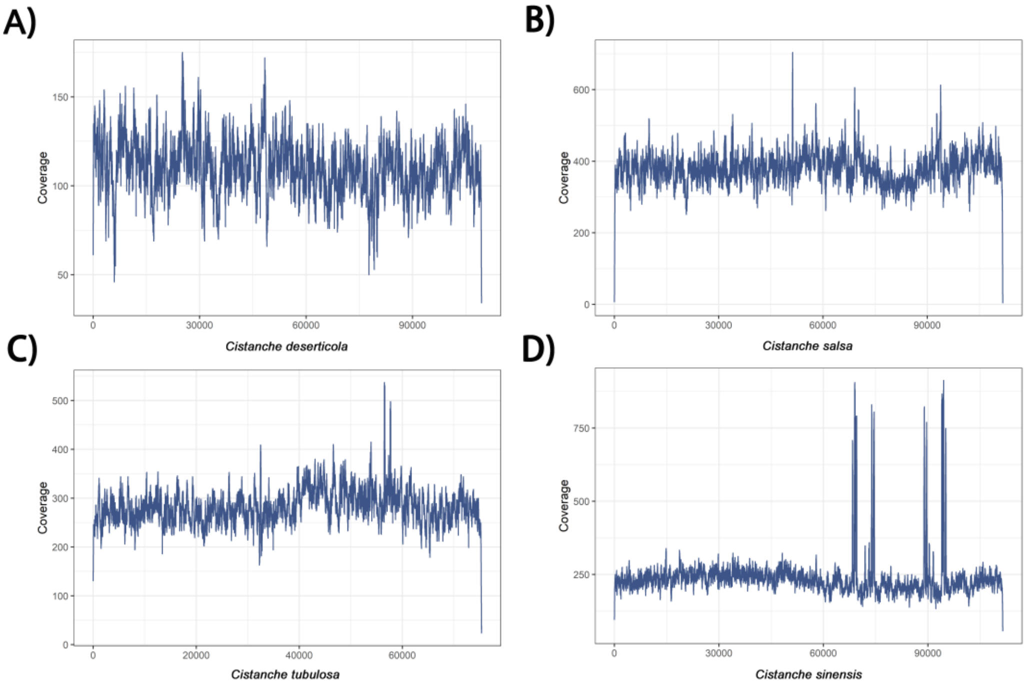

Fig. S1. The coverage depth of the four *Cistanche* plastomes. The raw sequence reads were mapped to the reference plastome sequences. A) *C. deserticola*; B) *C. salsa*; C) *C. sinensis*; D) *C. tubulosa*. The X-axis shows the plastome positions. The Y-axis shows the depth. The Y-axis shows the coverage depth of the mapped reads.

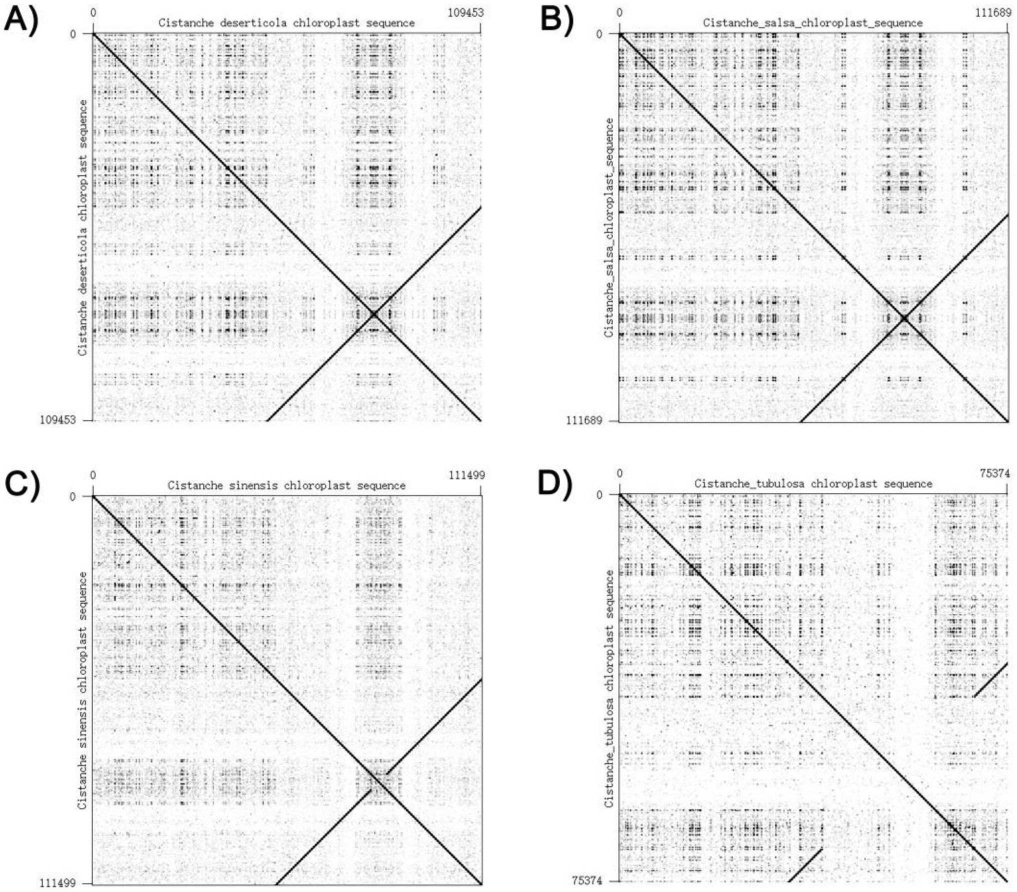

Fig. S2. The dot plots showing the self-2-self alignment of the four *Cistanche* plastomes sequences. The plots were generated using Gepard. A) *C. deserticola*; B) *C. salsa*; C) *C. sinensis*; D) *C. tubulosa*.

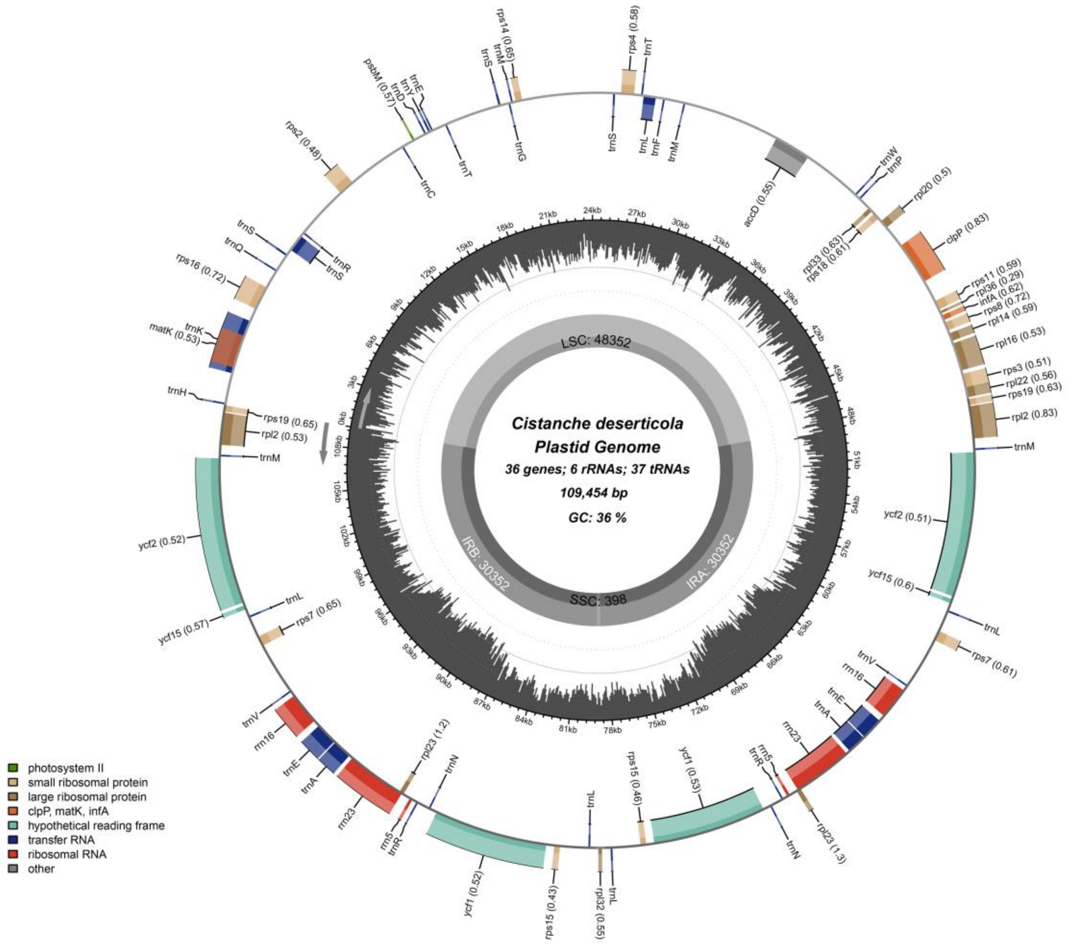

Fig. S3. A schematic map of the *Cistanche deserticola* plastome. The first circle shows the species name and specific information regarding the genome (length, GC content, and the number of genes) from the center going outward. The second circle shows the length of the corresponding single short copy (SSC), inverted repeat (IRa and IRb), and large single-copy (LSC) regions from the center going outward. The third circle shows the GC content. The outer circle shows the gene names and their optional codon usage bias in parentheses. The genes are colored based on their functional categories. Genes inside and outside of the circle are transcribed in clockwise and counterclockwise directions, represented with arrows.

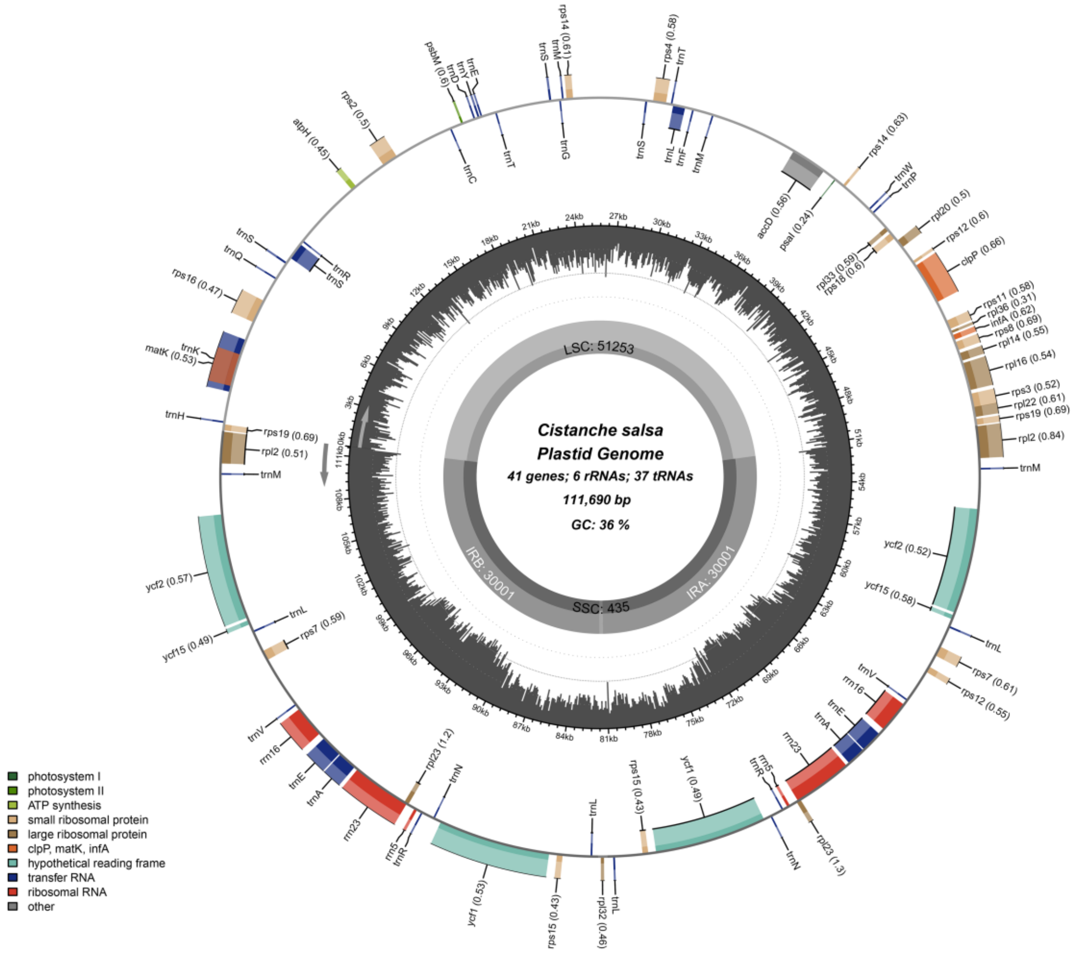

Fig. S4. A schematic map of the *Cistanche salsa* plastome. The first circle shows the species name and specific information regarding the genome (length, GC content, and the number of genes) from the center going outward. The second circle shows the length of the corresponding single short copy (SSC), inverted repeat (IRa and IRb), and large single-copy (LSC) regions from the center going outward. The third circle shows the GC content. The outer circle shows the gene names and their optional codon usage bias in parentheses. The genes are colored based on their functional categories. Genes inside and outside of the circle are transcribed in clockwise and counterclockwise directions, represented with arrows.

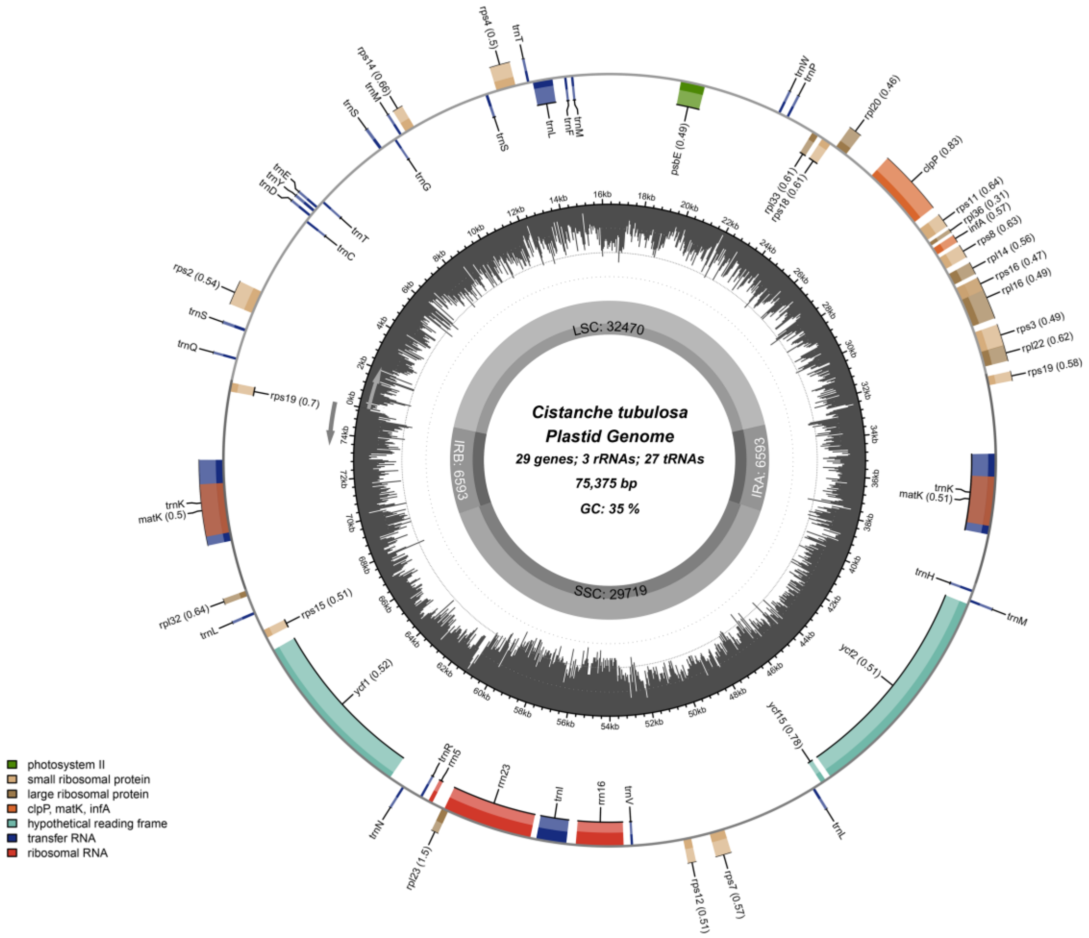

Fig. S5. A schematic map of the *Cistanche tubulosa* plastome. The first circle shows the species name and specific information regarding the genome (length, GC content, and the number of genes) from the center going outward. The second circle shows the length of the corresponding single short copy (SSC), inverted repeat (IRa and IRb), and large single-copy (LSC) regions from the center going outward. The third circle shows the GC content. The outer circle shows the gene names and their optional codon usage bias in parentheses. The genes are colored based on their functional categories. Genes inside and outside of the circle are transcribed in clockwise and counterclockwise directions, represented with arrows.

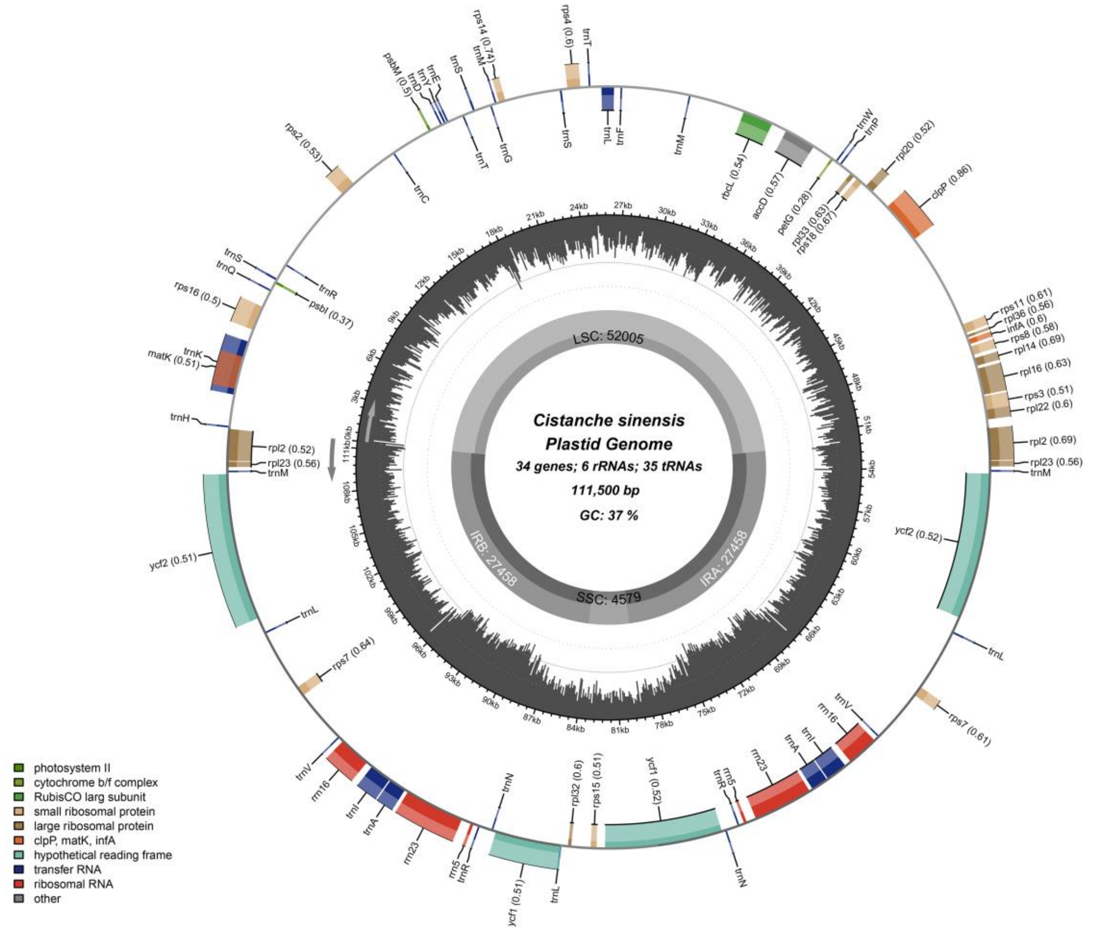

Fig. S6. A schematic map of the *Cistanche sinensis* plastome. The first circle shows the species name and specific information regarding the genome (length, GC content, and the number of genes) from the center going outward. The second circle shows the length of the corresponding single short copy (SSC), inverted repeat (IRa and IRb), and large single-copy (LSC) regions from the center going outward. The third circle shows the GC content. The outer circle shows the gene names and their optional codon usage bias in parentheses. The genes are colored based on their functional categories. Genes inside and outside of the circle are transcribed in clockwise and counterclockwise directions, represented with arrows.

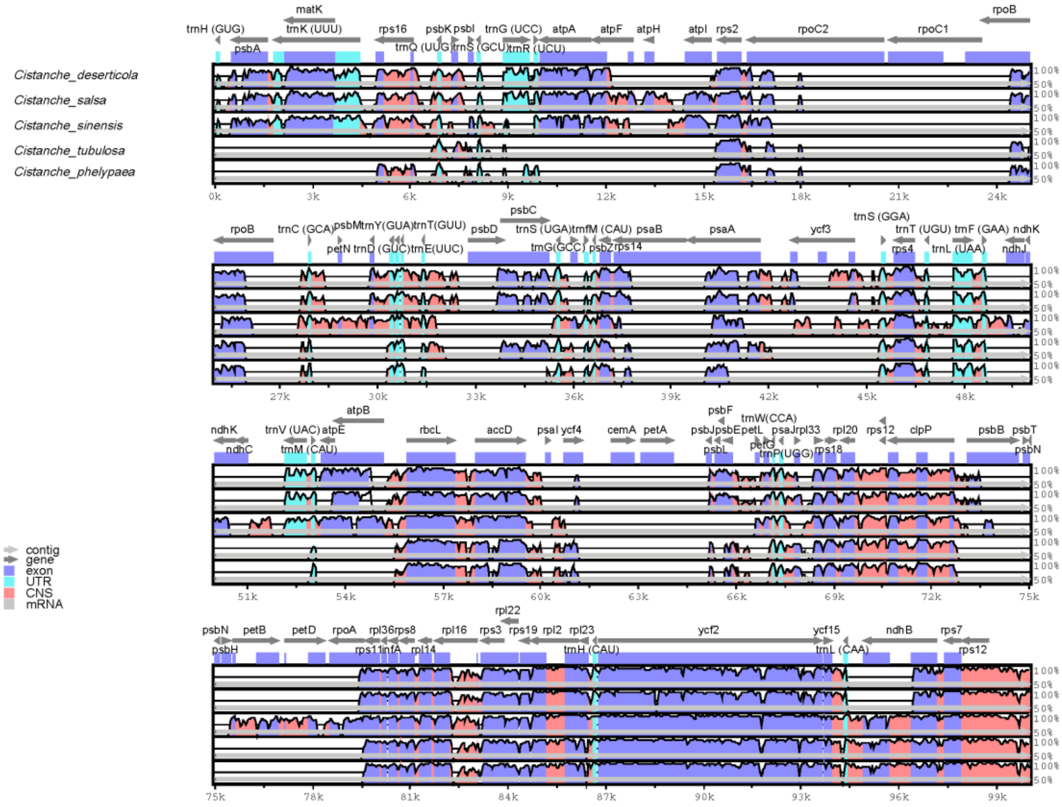

Fig. S7. Identity plot comparing the plastid genomes of *C. deserticola*, *C. salsa*, *C. sinensis*, *C. tubulos*a and *C. phelypaea* using *Rehmannia glutinosa* as a reference sequence. The vertical scale indicates the percentage of identity (50% to 100%), using a 50% identity cutoff. The horizontal axis indicates the coordinates in the plastomes. Genomeregions are color-coded as protein-coding, rRNA, tRNA, intron, and conserved non-coding sequences (CNS).

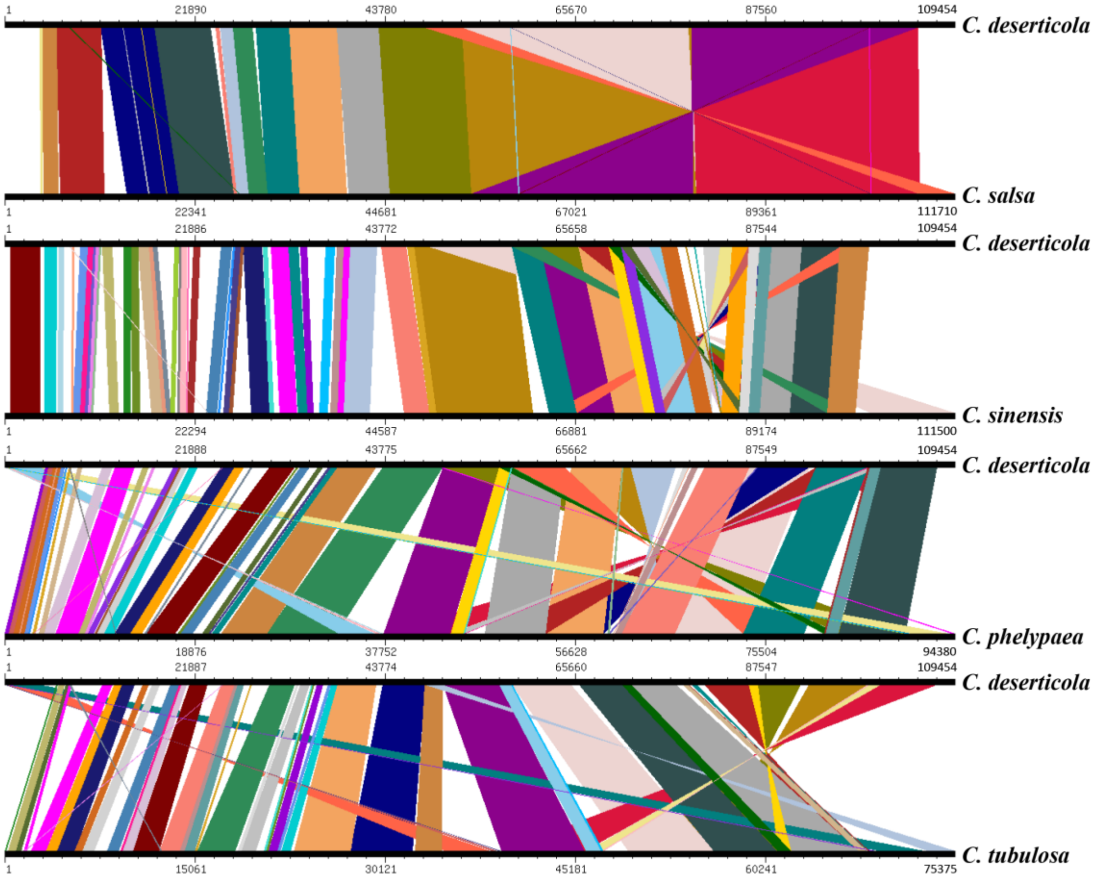

Fig. S8. Synteny analyses of plastomes for five *Cistanche* species. Each horizontal black line represents a genome, with conserved regions connected with colored blocks. The plastome sequence of *C. deserticola* was used as the reference.

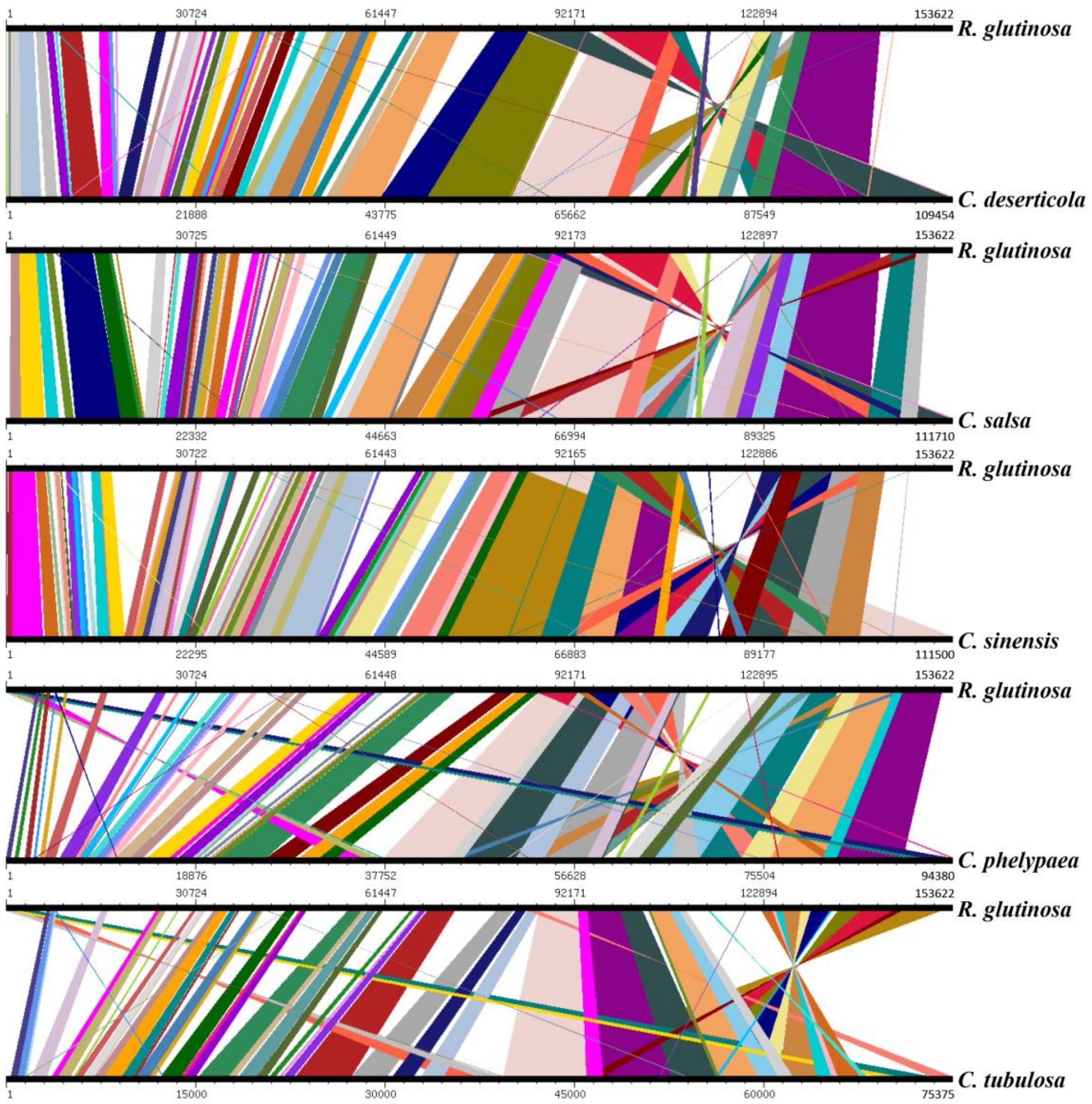

Fig. S9. Synteny analyses of five *Cistanch*e plastomes compared with *Rehmannia glutinos*a. Each horizontal black line represents a genome, with conserved regions connected with colored block. The plastome of *R. glutinosa* was used as the reference.

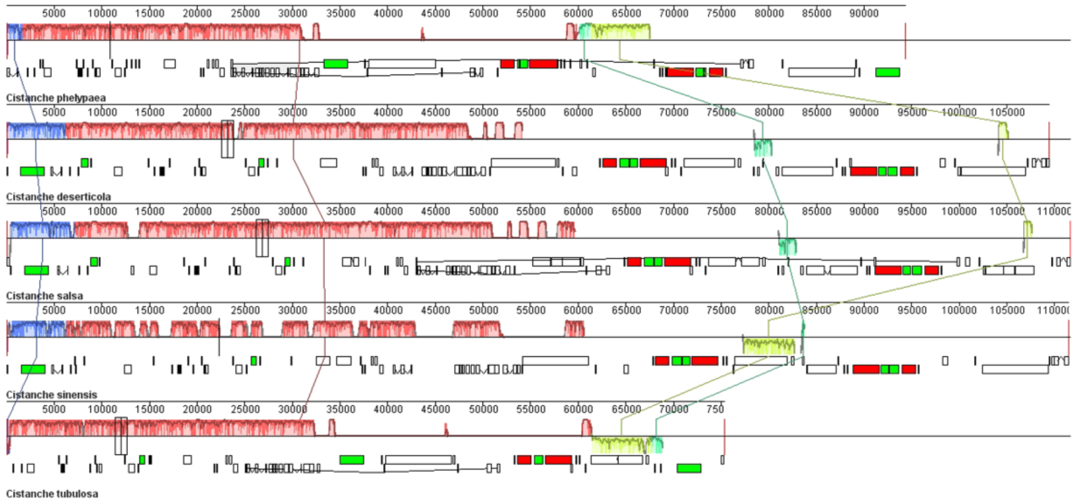

Fig. S10. Extent of the gene rearrangements of 5 *Cistanche* plastomes. Locally collinear blocks of the sequences are colour-coded and connected by lines.

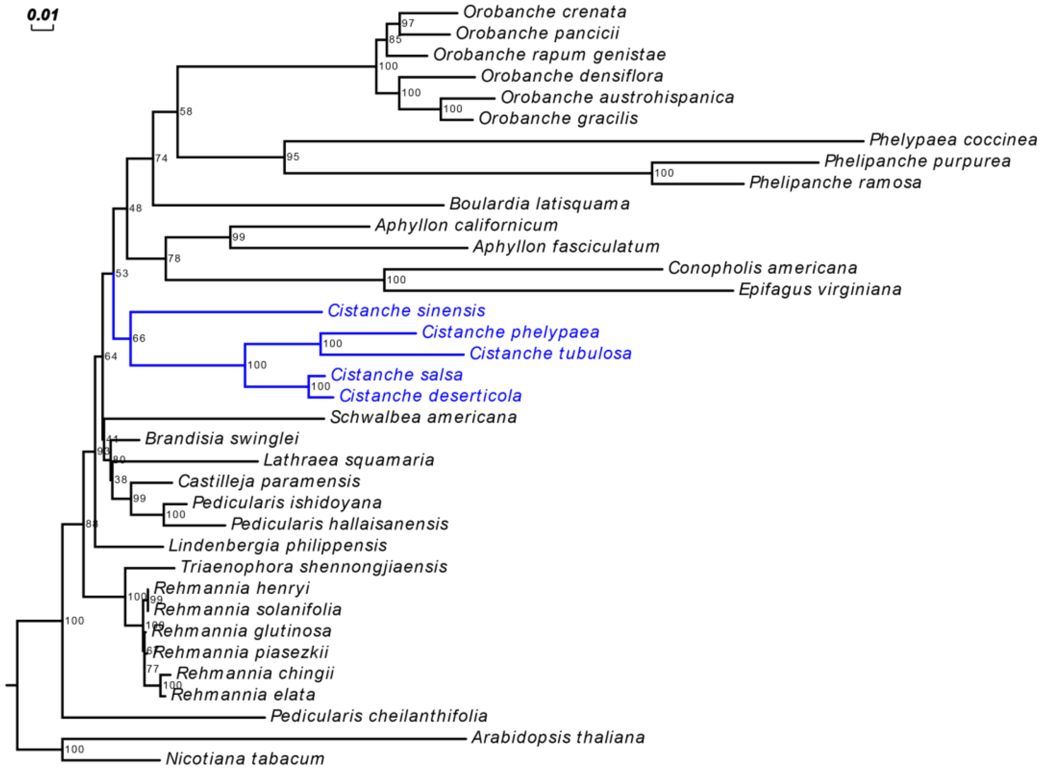

Fig. S11. Maximum likelihood (ML) Phylogenetic tree of 36 *Orobanchaceae* species. The *Cistanche* species are highlighted in blue. The bootstrap scores are shown on the corresponding branches. The detail information can be found in Table S2.

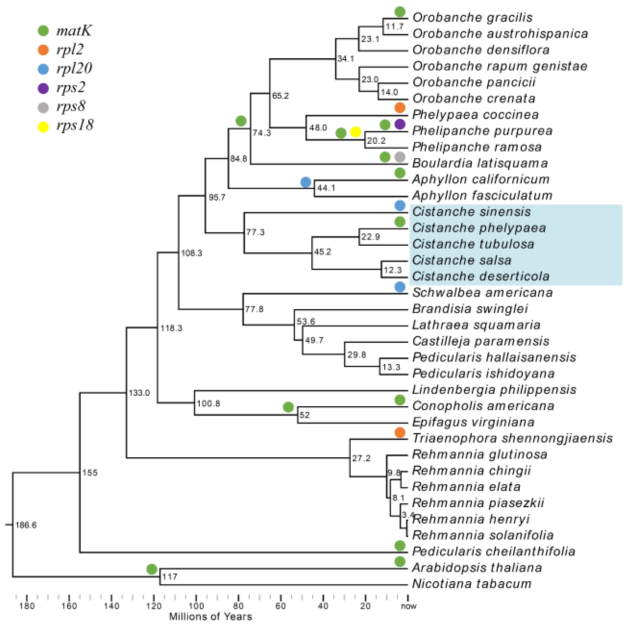

Fig. S12. Maximum clade credibility tree obtained from a molecules clock analysis using the BEAST software. The circles having different colors represent the genes positively selected in *Orobanchaceae*. The background of *Cistanche* is highlighted in lightblue. The detail information can be found in Table S2.

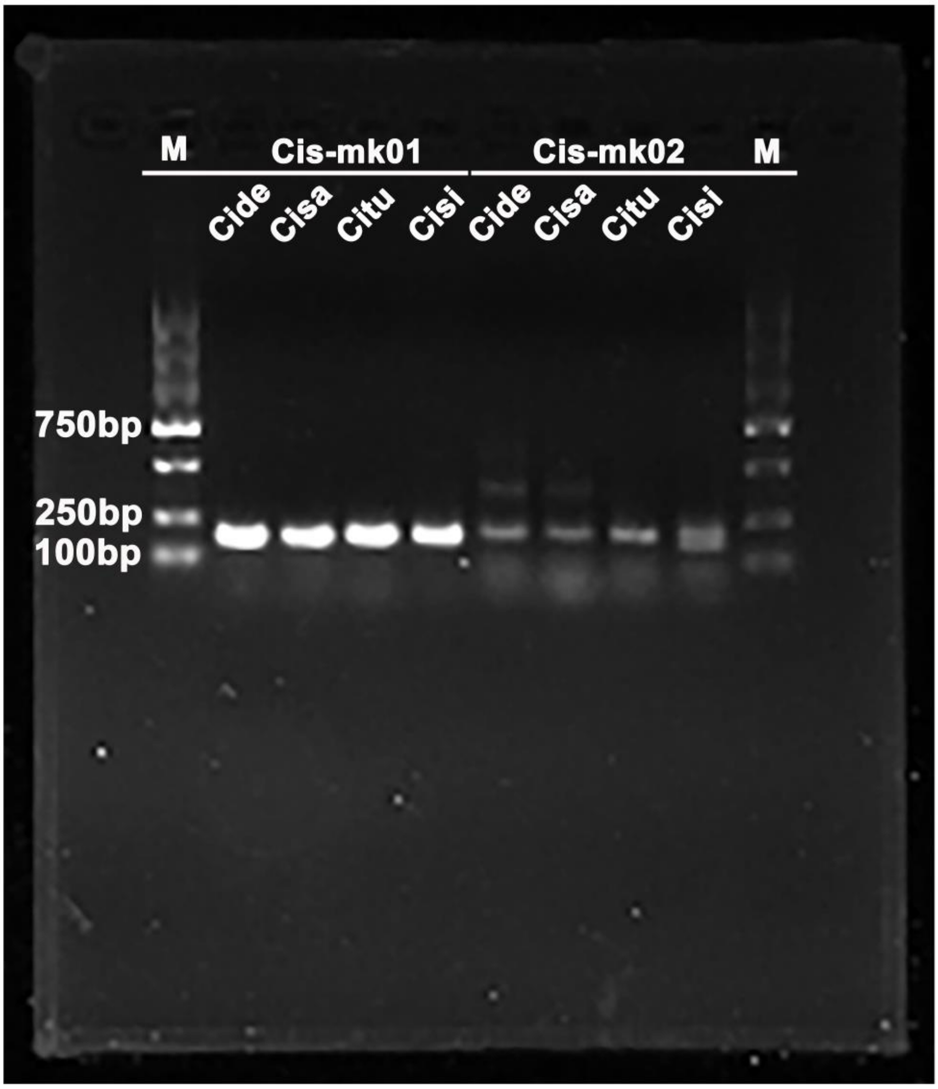

Fig. S13. The gel electrophoresis results of the PCR products amplified using the primers pairs list in Table S7 using DNA marker. Lane M was the marker of DL 2000. The lanes from left to right corresponded to products amplificated from the first individual of *C. deserticola* (Cide), *C. salsa* (Cisa), *C. tubulosa*(Citu), and *C. sinensis* (Cisi) by primer Cis-pp01 and Cis-pp02, respectively.

A)

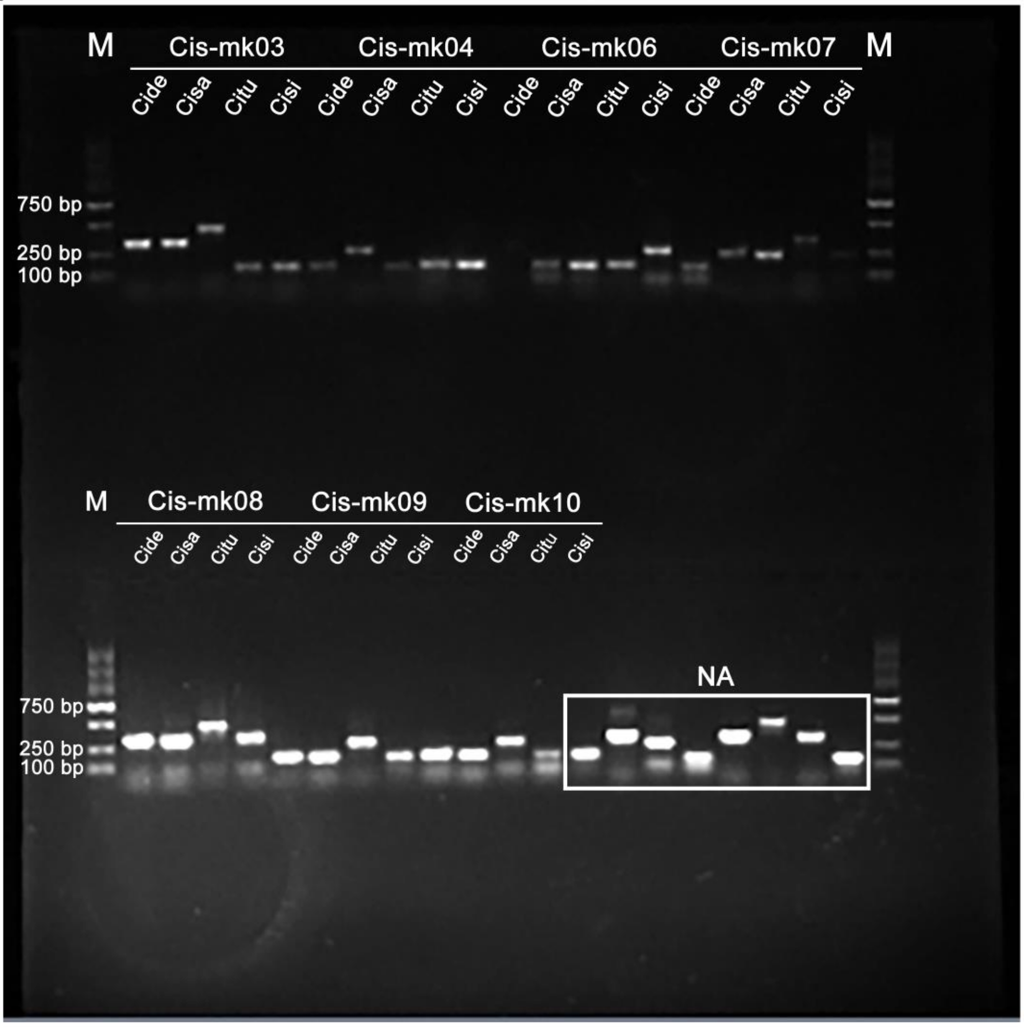
M
Cis-mk03
Cis-mk04
Cis-mk06
Cis-mk07
M
Cide Cisa Citu Cisi Cide Cisa Citu Cisi Cide Cisa Citu Cisi Cide Cisa Citu Cisi
750 bp
250 bp
100 bp
M
Cis-mk08
Cis-mk09
Cis-mk10
Cide Cisa Citu Cisi Cide Cisa Citu Cisi Cide Cisa Citu Cisi
NA
750 bp
250 bp
100 bp

B)

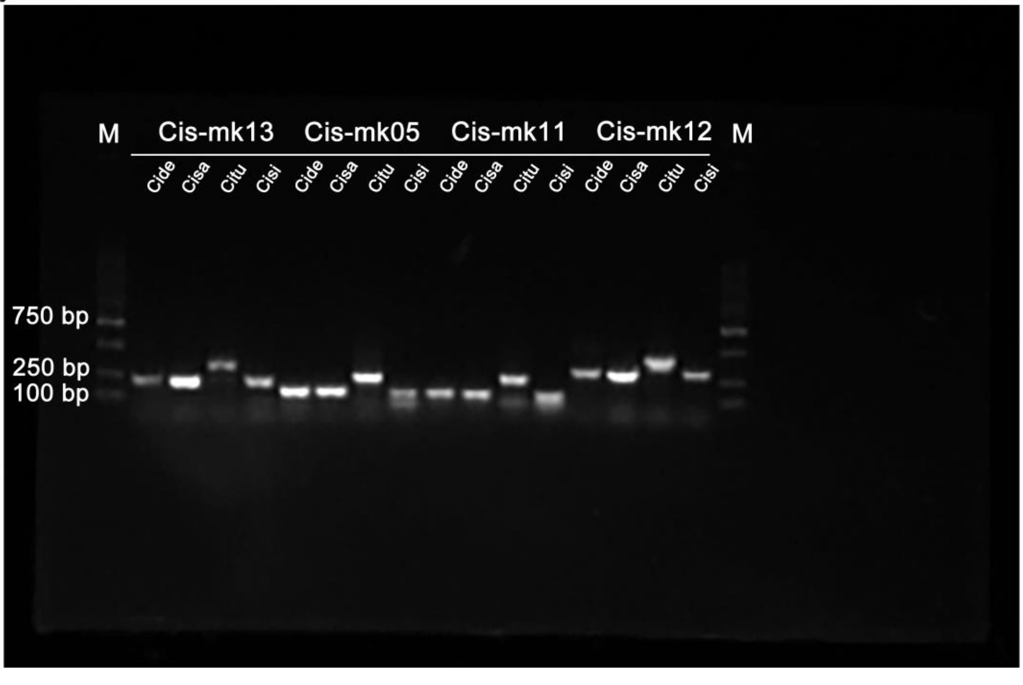
M
Cis-mk13
Cis-mk05
Cis-mk11
Cis-mk12
M
Cide Cisa Citu Cisi Cide Cisa Citu Cisi Cide Cisa Citu Cisi Cide Cisa Citu Cisi
750 bp
250 bp
100 bp

Fig. S14. The gel electrophoresis results of the PCR products amplified using the primers pairs list in Table S7 using DNA marker. Lane M was the marker of DL 2000. The lanes from left to right corresponded to products amplificated from the first individual of *C. deserticola* (Cide), *C. salsa* (Cisa), *C. tubulosa* (Citu), and C. *sinensis* (Cisi) by primer Cis-pp03 to Cis-pp013, respectively.

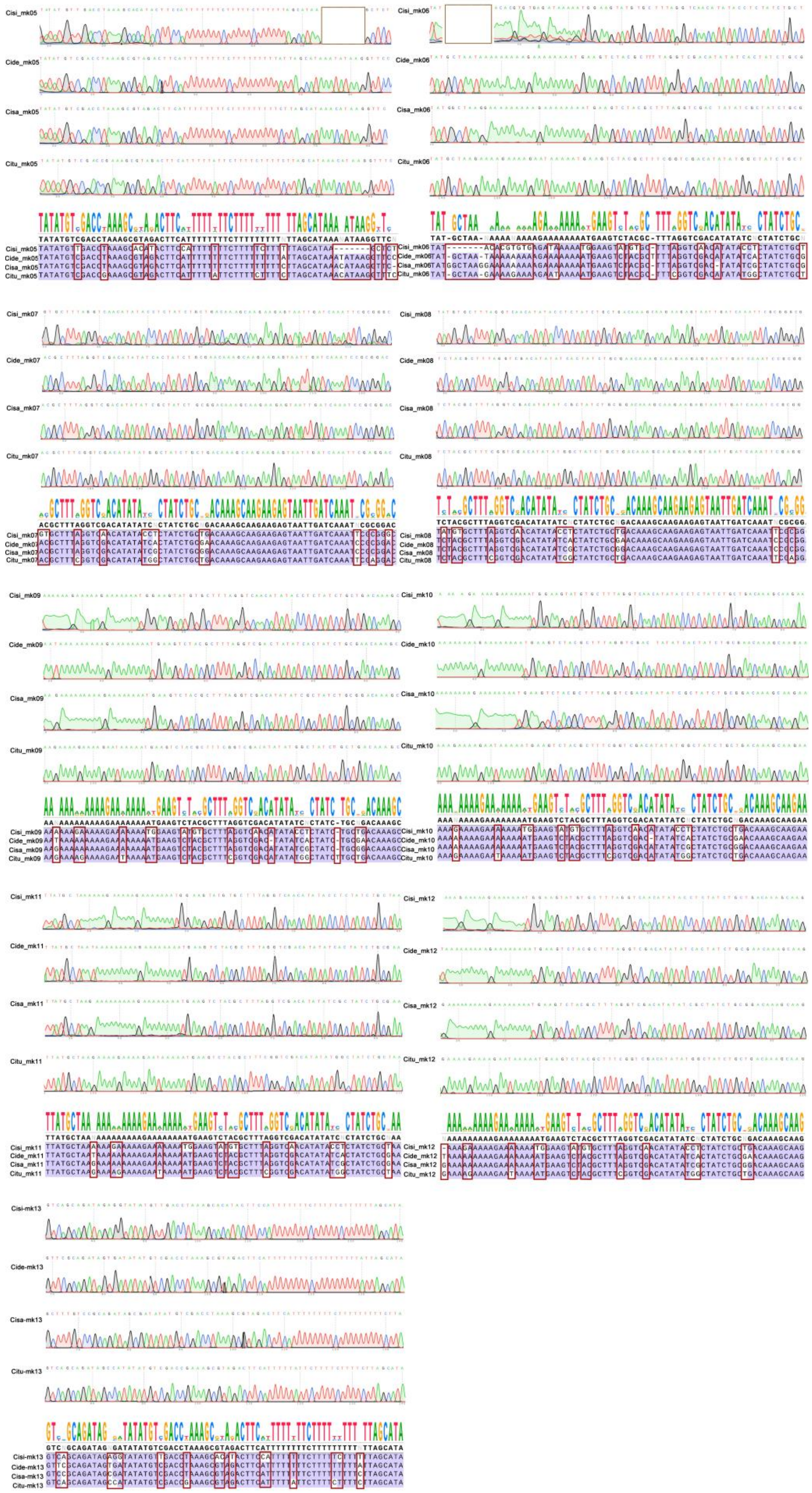
Cisi_mk05
Cide_mk05
Cisa_mk05
Citu_mk05
Cisi_mk06
Cide_mk06
Cisa_mk06
Citu_mk06
Cisi_mk07
Cide_mk07
Cisa_mk07
Citu_mk07
Cisi_mk08
Cide_mk08
Cisa_mk08
Citu_mk08
Cisi_mk09
Cide_mk09
Cisa_mk09
Citu_mk09
Cisi_mk10
Cide_mk10
Cisa_mk10
Citu_mk10
Cisi_mk11
Cide_mk11
Cisa_mk11
Citu_mk11
Cisi_mk12
Cide_mk12
Cisa_mk12
Citu_mk12
Cisi-mk13
Cide-mk13
Cisa-mk13
Citu-mk13

Fig. S15. The alignment of the sequencing chromatogram of the PCR products amplified using DNA marker (Cis-mk03 to Cis-mk13). SNP and Indel regions were highlighted with red squares. Cide: *C. deserticola*; Cisa: *C. salsa*; Citu: *C. tubulosa*; Cisi: *C.sinensis*.
